# Empagliflozin Mitigates PTZ-Induced Seizures in Rats: Modulating Npas4 and CREB-BDNF Signaling Pathway

**DOI:** 10.1007/s11481-024-10162-6

**Published:** 2025-01-07

**Authors:** Heba A Abdelaziz, Mohamed F. Hamed, Hamdy A. Ghoniem, Manar A. Nader, Ghada M. Suddek

**Affiliations:** 1 Pharmacology and Biochemistry Department, Faculty of Pharmacy, Delta University for Science and Technology, Gamasa, 35712 Egypt; 2 Pharmacology and Toxicology Department, Faculty of Pharmacy, Mansoura University, Mansoura, 35516 Egypt; 3 Pathology Department, Faculty of Veterinary Medicine, Mansoura University, Mansoura, 35516 Egypt; 4 Pharmacology and Toxicology Department, Faculty of Pharmacy, Mansoura National University, Gamasa, 7731168 Egypt

**Keywords:** Empagliflozin, PTZ, BDNF, CREB, Npas4, Neuroplasticity

## Abstract

Empagliflozin (EMPA) is one of the sodium/glucose cotransporter 2 (SGLT2) inhibitors that has been recently approved for the treatment of diabetes mellitus type II. Recently, EMPA has shown protective effects in different neurological disorders, besides its antidiabetic activity. Kindling is a relevant model to study epilepsy and neuroplasticity. This study aimed to investigate the potential protective effects of EMPA (1 and 3 mg/kg orally) against convulsant effects induced by pentylenetetrazole (PTZ) using a modified window- (win-) PTZ kindling protocol. The biochemical dysfunction and hippocampal damage induced by PTZ were profoundly reversed by EMPA treatment in a dose-dependent manner, as evidenced by the significant increase in reduced glutathione (GSH) and decrease in malondialdehyde (MDA) hippocampal contents. Furthermore, EMPA counteracted PTZ-induced neuronal damage in the hippocampal region, as confirmed by histopathological examination of the hippocampal tissues. EMPA impaired astrocytosis and showed an antiapoptotic effect through a significant reduction of glial fibrillary acidic protein (GFAP) and BCL2-Associated X Protein (BAX) expressions, respectively. Interestingly, EMPA exhibited an antiepileptic effect against PTZ-induced seizures through significantly reducing neuronal PAS domain Protein 4 (Npas4), cyclic adenosine monophosphate (cAMP) response element binding protein (CREB) hippocampal expressions, and enhancing the brain-derived neurotrophic factor (BDNF)-tropomyosin receptor kinase B (TrkB) pathway, which are found to be involved in epileptogenesis, eventually leading to significant improvement of behavioral impairments induced by PTZ. Hence, these results showed further prospective insights for EMPA as a neuroprotective agent.

**Graphical Abstract:**

**Figure:**
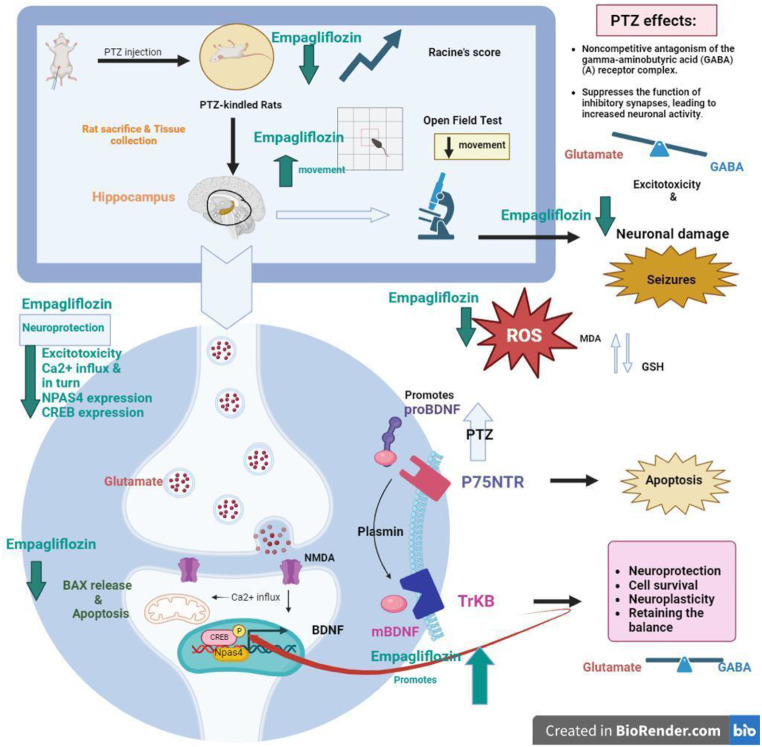
The effect of pretreatment with EMPA (1&3 mg/kg) against PTZ induced kindling epilepsy in rats. * PTZ was intraperitoneally injected (37.5 mg/kg) where rats received only seven injections: 4 initial doses every other day followed by 3 consecutive injections at 29^th^, 31^st^ and 33^rd^ days to induce seizures in rats. EMPA pretreatments were given at 1 and 3 mg/kg orally daily. Abbreviations: PTZ: pentylenetetrazole, EMPA: empagliflozin, win-: window

**Supplementary Information:**

The online version contains supplementary material available at 10.1007/s11481-024-10162-6.

## Introduction

Epilepsy is one of the most common neurological disorders that affects about 1–3% of the population. It is characterized by the recurrence of unprovoked seizures spontaneously (Shneker and Fountain 2003). In spite of the upcoming developments in studying epilepsy and trials for its management, about 20% of patients are resistant to treatment (Erdogan et al. 2018). Therefore, new trends and research in epilepsy have been pronounced, seeking to reduce the frequency and severity of this debilitating illness. As well, there is an urge to introduce new strategies to prevent epilepsy development (epileptogenesis) rather than just predominantly symptomatic treatments that only control seizures (French et al. 2021).

Recent studies have focused on studying the brain-derived neurotrophic factor (BDNF) pathway and its role in epileptogenesis (Hughes et al. 1999); (Scharfman 2005); (Wang et al. 2021). In the PTZ-induced epilepsy model, the upregulation of BDNF suggests its role in the enhancement of glutamatergic response and hindering GABAergic neurotransmission (Nader et al. 2018). However, BDNF is suggested to show anti-epileptic effects in other studies (Cifelli et al. 2013); (Sharma et al. 2020). BDNF is a member of the neurotrophin family, where the precursor (proBDNF) undergoes proteolytic cleavage into the mature BDNF (mBDNF) through the tissue-type plasminogen activator (tPA)-plasmin (Tsai 2007); (Wang et al. 2021). Recently, studies indicated that the relative ratio of mature BDNF to its precursor is considered a differential diagnostic biomarker for distinguishing different pathological disorders (Zhao et al. 2017); (Dorandish et al. 2021). BDNF (mBDNF) binds to its receptor, tropomycin receptor kinase B (TrkB), where the mBDNF/TrkB receptor complex potentiates neuronal survival and neuroplasticity. On the contrary, proBDNF binds to the p75 NGF receptor (p75NTR), which aggravates apoptosis (Je et al. 2013); (Pang et al. 2016).

Among the mediators of BDNF transcription are neuronal PAS domain Protein 4 (Npas4) (Fu et al. 2020) and cyclic adenosine monophosphate (cAMP) response element binding protein (CREB) family transcription factors (Esvald et al. 2020). Npas4 is an immediate early gene (IEG) that is selectively induced by calcium influx only in neurons and is mediated in response to excitatory transmission (Sun and Lin 2016); (Lissek et al. 2021), where its expression triggers the transcriptional activity of other factors such as c-Fos and BDNF (Funahashi et al. 2019). It is considered one of the first neuroplastic changes that plays an essential role in neuroplastic events and the management of different neurological disorders, including epilepsy (Jarero-Basulto et al. 2018). Npas4 is thought to exert its functions through the regulation of BDNF expression, where BDNF plays a central role in inhibitory synapse formation, neuroplasticity, and neuroprotection (Colucci-D’Amato et al. 2020). Accordingly, Npas4 seems to play a role in the reduction of neuronal activity levels and promotion of GABA-mediated inhibitory transmission, so it may function as a homeostatic mechanism during processes of excitability (Maya-Vetencourt 2013). Therefore, Npas4 acts via a self-regulating negative feedback mechanism through BDNF regulation (Lin et al. 2008). Recent studies revealed the potential role of the Npas4 transcription factor in restoring and regulating plasticity, and it has been considered a novel therapeutic target for different neurological disorders and future drug development (Maya-Vetencourt et al. 2012). CREB is also considered one of the transcriptional factors that regulate BDNF transcription, where the active phosphorylated CREB (pCREB) binds to a specific site in the BDNF promoter (Wang et al. 2018). At the same time, BDNF was found to induce CREB phosphorylation through the mitogen-activated protein kinase (MAPK) pathway in a mutual relationship (Pizzorusso et al. 2000). Like Npas4, CREB is regulated by Ca2 + influx (Kornhauser et al. 2002); therefore, calcium signaling was also found to contribute either directly or indirectly to epileptogenesis (Steinlein 2014).

The PTZ-kindling model is one of the most widely studied chronic models that mimic many features of human epilepsy (Ngoupaye et al. 2022) and the epileptogenesis process (Hughes et al. 1999). In the PTZ-kindling model, seizures initiate a series of deleterious effects, including oxidative stress as indicated by alterations in oxidative biomarkers (Łukawski and Czuczwar 2023), leading to neuronal loss, gliosis (Carmody and Brennan 2010), and apoptosis (Bibi et al. 2017). In addition, hippocampal damage (Gao et al. 2018) and behavioral changes (Erdoğan et al. 2005) are attributed.

Some epileptic patients are resistant to the current epilepsy therapies that are directed at altering the function of neurotransmitter receptors, ion channels, or even the release of synaptic vesicles (Zhu et al. 2015). Therefore, metabolism-based therapies have been recently studied as novel targets to prevent seizures rather than the traditional anti-inflammatory drugs and the inhibitory drugs that act at the synaptic level (Rho and Boison 2022). Sodium-glucose co-transporter 2 (SGLT2) receptors are found in the proximal tubules of the kidneys, where they mediate glucose reabsorption from the urine. Therefore, the inhibition of SGLT2 is a novel strategy that is currently used for the management of diabetes mellitus (DM) (Norton et al. 2017). SGLTs are also found to be widely expressed in the brain and to play an essential role in glucose utilization (Yu et al. 2013). Surprisingly, SGLT2i are lipid-soluble drugs with the ability to cross the blood-brain barrier (BBB) (Pawlos et al. 2021). EMPA, one of the FDA-approved SGLT2i, acts independently of insulin by lowering glucose availability through increasing its excretion and decreasing its absorption in the proximal tubules in the kidney (Sizar et al. 2022). Besides its antidiabetic activity, EMPA has recently shown potent anti-inflammatory, anti-oxidant, and neuroprotective effects in different neurological disorders, including Parkinson’s disease (PD) (Ahmed et al. 2022), Alzheimer’s disease (Hierro-Bujalance et al. 2020), and cereberal ischemia (Amin et al. 2020). Moreover, SGLT2 inhibitors are thought to reduce seizures induced by PTZ in rats, suggesting their stabilizing effects against excitation and epileptic discharges (Erdogan et al. 2018). Based on the aforementioned studies, we were motivated in this experimental model to investigate the possible protective effects of EMPA pretreatment (1 and 3 mg/kg) against PTZ-induced seizures in rats using the win-PTZ kindling method and to figure out the mechanism that may underlie its antiepileptic effect and the implication of the BDNF pathway in an attempt to provide a new therapeutic venue for EMPA as a neuroprotective drug that may target the process of epileptogenesis.

## Materials & Method

### Animals

A total of thirty male Sprague-Dawley rats, weighing 200–250 g, were selected for the study. They were purchased from the animal house at Delta University of Science and Technology, where free food and water were provided *ad libitum*. All medical care and handling procedures met the ethical committee guidelines (Faculty of Pharmacy, Mansoura University).

### Chemicals and Drugs

PTZ (37.5 mg/kg, i.p.) was purchased from Sigma Aldrish Co., USA Sodium valproate (200 mg/kg, i.p.) was obtained from Delta Pharma Co., Egypt. Empagliflozin tablets (Jardiance^®^ 10 mg, Boehringer Ingelheim, Germany) were disintegrated and suspended in 0.5% carboxymethylcellulose (CMC). GSH and MDA-available kits were purchased from Biodiagnostic Co. (Giza, Cairo). BDNF and CREB antibodies were purchased from Affinity Biosciences Co., USA. BAX polyclonal antibody was purchased from ABclonal^®^ Co., USA, and GFAP polyclonal antibody was purchased from Diagnostic Biosystems Co., USA. The Npas4 ELISA kit was purchased from Bioassay Technology Laboratory (Shanghai, China).

### Experimental Model and Specimen Collection

**Fig. 1:**
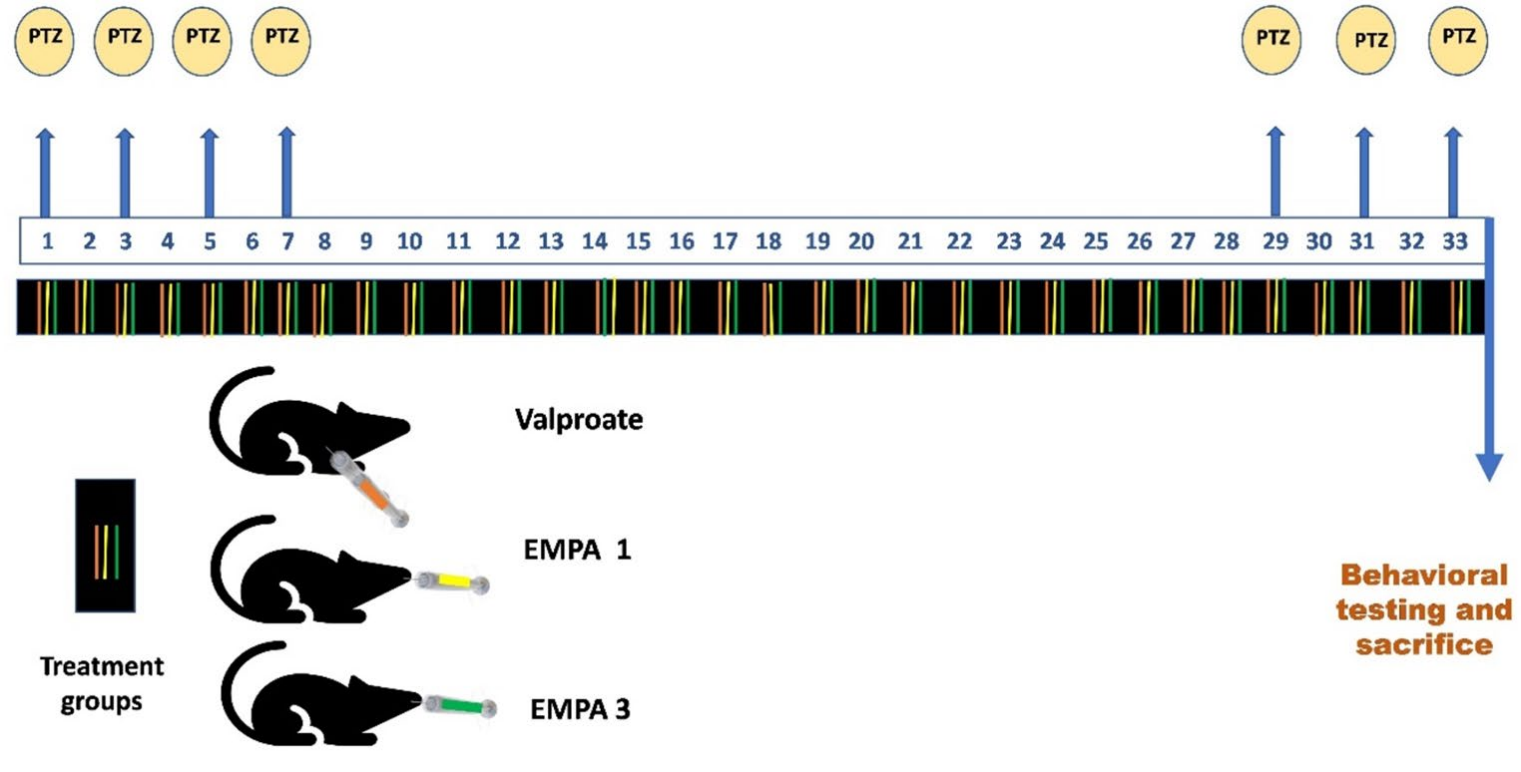
Experimental design showing days of injections of (**a**) PTZ, (**b**) VPA, and (**c**) EMPA 1 mg/kg & 3 mg/kg. * PTZ was intraperitoneally injected (37.5 mg/kg) where rats received only seven injections: 4 initial doses day after day followed by 3 consecutive injections at the 29^th^, 31^st^, and 33^rd^ days to induce seizures in rats. VPA pretreatment was given at 200 mg/kg i.p. daily. EMPA pretreatments were given at 1 and 3 mg/kg orally daily. Abbreviations: PTZ: pentylenetetrazole, VPA: valproate, EMPA: empagliflozin, win-: window

A modified protocol named the win-PTZ kindling method has been established, in which animals received only four initial intraperitoneal (i.p.) injections of PTZ (37.5 mg/kg) every other day, followed by a time window where the animals didn’t receive PTZ during the next 22 days, and thus the three last PTZ injections were done at the 29^th^, 31^st^, and 33^rd^ days. This model is considered a less invasive method with less application of PTZ injections than the standard PTZ kindling model of epilepsy for the induction of the fully kindled state in animals (Davoudi et al. 2013). Animals were randomized into five groups as follows (Fig. 1): Group 1: control group (*n* = 6), where rats received normal saline i.p. daily; Group 2: PTZ group (*n* = 6), rats received only seven injections, where they received 4 initial PTZ injections (37.5 mg/kg i.p.) every other day followed by 3 final injections at the 29^th^, 31^st^, and 33^rd^ days; Group 3: VPA group (*n* = 6), where rats received 200 mg/kg i.p. daily 10.1523/JNEUROSCI.2506-11.2011. PMID: 22072692; PMCID: PMC6633222 and 30 min prior to PTZ injections; Group 4: EMPA (1 mg/kg) (*n* = 6), where rats received 1 mg/kg orally daily and 30 min prior to PTZ injections; and Group 5: EMPA (3 mg/kg) (*n* = 6), where rats received 3 mg/kg orally daily and 30 min prior to PTZ injections. The two doses of EMPA were selected after dose conversion of the two recommended clinical doses of EMPA (10 mg & 25 mg daily) (Matsumura et al. 2022) according to Nair’s practice guide (Nair and Jacob 2016) and were administered orally by gastric gavage using an orogastric tube to ensure complete ingestion (Turner et al. 2012). On day 34, behavioral tests were performed to assess locomotor and mental activities. The hippocampus is considered the most studied and most vulnerable region to seizures in epilepsy (McEwen 1994), so the animals were sacrificed, where the brain was preserved in ice-cold saline to be dissected into two hemispheres with left and right hippocampi. One hippocampal hemisphere was homogenized using 0.05 M phosphate buffer for oxidant/antioxidant status assessment, western blotting, and enzyme-linked immunosorbent assay (ELISA). After dilution, the correction factor equation was applied to extrapolate the results to the original undiluted values, according to the manufacturer’s instructions. The other hippocampal section was fragmented into thin sections and kept in formalin for the preparation of paraffin-embedded blocks of tissue to be sent for histopathology and immunohistochemistry examination. Other ultrathin sections were fixed in 2.5% v/v glutaraldehyde to be examined using an electron microscope.

### Seizure Score

The rats were placed in a box where behavioral changes were monitored within a period of 20 min following PTZ injections and scored according to Racine’s stages scores (RSS) (Corda et al. 1990) as follows:

0: for no response.

1: for facial and ear twitches.

2: for myoclonic jerks.

3: for myoclonic jerks with rearing.

4: for clonic convulsions with turning over into side position.

5: generalized tonic-clonic seizures or death.

According to Davoudi et al.‘s study, a few animals may show generalized seizures or death during the first four injections, and if so, they have to be excluded from the experiment.

### Behavioral Test: Open Field Test

The open-field test has recently been widely used to serve as a tool to assess the locomotor and exploratory impairments associated with different CNS disorders as well as the anxious-like side effects in rodents (Shariare et al. 2020). A box that is divided into 16 squares and surrounded by high walls was used (Valvassori et al. 2017) where;

- latency (time to start moving),
- number of squares crossed with four legs,
- groomings,
- and rearings were recorded.

The rats were placed gently in the center of the box, and their behaviors were recorded using a digital camera (Canon EOS 250D, Japan) over 5 min for data analysis. The animals should be initially habituated to the test, and the test box should be cleaned thoroughly using an ethanol solution each time and especially between groups (de Souza et al. 2019).

## Histopathological Examination

### Light Microscope Examination Using Hematoxylin and Eosin (H & E) Stains

Hippocampal sections were isolated and fixed using 10% neutral buffered formalin for paraffin block preparation. Serial sagittal sections were stained using H&E stains and examined by a light microscope. Neuronal cell counts for intact pyramidal cells in the CA3 area, as well as the thickness (µm), were photographed and conducted using the Olympus^®^ microscope (CX43) and its linked software, with three fields from each animal per group per slide at high power field (HPF) (El-Adli et al. 2023).

### Transmission Electron Microscope (TEM) Examination

Ultrathin sections were initially fixed using glutaraldehyde, then post-fixed in 2% osmium tetraoxide at 0.1 M, pH 7.4 phosphate buffer to be further stained with uranyl acetate and lead citrate and examined by TEM (Yardımoğlu et al. 2007). All prepared slides were examined blindly by the reader.

### Hippocampal Immunohistochemical Examination

Briefly, the samples were deparaffinized, then rehydrated with subsequent antigen retrieval. Hydrogen peroxide solution (H_2_O_2_ 3%) was added to the slides to prevent endogenous peroxidase activity, followed by primary antibodies (anti-BAX 1:100) (ABclonal^®^, USA) and (anti-GFAP 1:100) (Diagnostic BioSystems, USA) to be incubated with the slides overnight. The slides were then rinsed three times using phosphate buffered saline (PBS) and incubated with the secondary antibody to be finally counterstained with hematoxylin and investigated using a light microscope according to the manufacturer’s instructions. The positively stained cells were counted per slide using the Olympus^®^ microscope (CX43) at HPF (400x).

## Assessment of NPAS4, BDNF, and CREB Contents

### Western Blot Analysis

Total hippocampal proteins were extracted using RIPA (radioimmunoprecipitation assay) buffer. Protein concentrations were determined by BCA (bicinchoninic acid) protein assay (Zhang et al. 2022). Then, the loading protein was mixed with the protein samples at a ratio of 1:1. The samples were electrophoresed on gradient sodium dodecyl sulfate-polyacrylamide gel electrophoresis (SDS-PAGE). After that, the proteins were blotted on a nitrocellulose membrane using transfer buffer. The membranes were blocked using 3% BSA (bovine serum albumin) to prevent non-specific interactions and were incubated overnight with the primary antibodies (anti-BDNF, Affinity Biosciences Co.) and (anti-CREB, Affinity Biosciences Co.) for at least sixty minutes. The membranes were rinsed thoroughly and finally incubated with the corresponding secondary antibody (horseradish peroxidase). The blots were visualized after the addition of 100 µL of TMB (tetramethylbenzidine) as a chromogenic substrate. The bands were quantitively analyzed according to relative densitometric analysis using ImageJ software (California, USA) after being normalized to the corresponding β-actin bands. The stripping technique was applied to allow the visualization of more than one protein using the same blot.

### Enzyme-Linked Immunosorbent Assay (ELISA)

Npas4 contents were evaluated in hippocampal sections using Bioassay Technology Laboratory ELISA kits (Shanghai, China) as described by the manufacturer.

### Elucidation of Oxidative Mechanism and Estimation of Stress Parameters

Hippocampal examination of non-enzymatic antioxidant (GSH) as well as lipid peroxidation aldehyde (MDA) contents was determined using pre-prepared Biodiagnostic Company kits (Giza, Egypt), following the mentioned procedures.

### Statistical Analysis

All parametric data were presented as mean ± SEM after being analyzed by one-way analysis of variance (ANOVA) test followed by (Tukey-Kramer) *post hoc* test. Seizure scores as non-parametric data were conducted as median ± interquartile range (IQR) using Friedman test, followed by Dunn’s *post hoc* test. All statistical data were calculated using GraphPad 9.3.1 software (GraphPad Software Inc., San Diego, CA, USA).

## Results

The treatment with EMPA (1 and 3 mg/kg) showed improvements in both behavioral and biochemical tests. However, pretreatment with EMPA at a higher dose (3 mg/kg) showed significant protection against PTZ-induced seizures rather than the lower one (1 mg/kg). VPA, as a standard antiepileptic drug, generally impacted the most neuroprotective effects with the highest recovery capacity.

### Effect of Pretreatment with VPA and EMPA (1&3 mg/kg) on Seizure Severity Based on Scoring and Behavioral Testing (Open Field Test) in win- PTZ Induced Kindling in rats

**Fig. 2:**
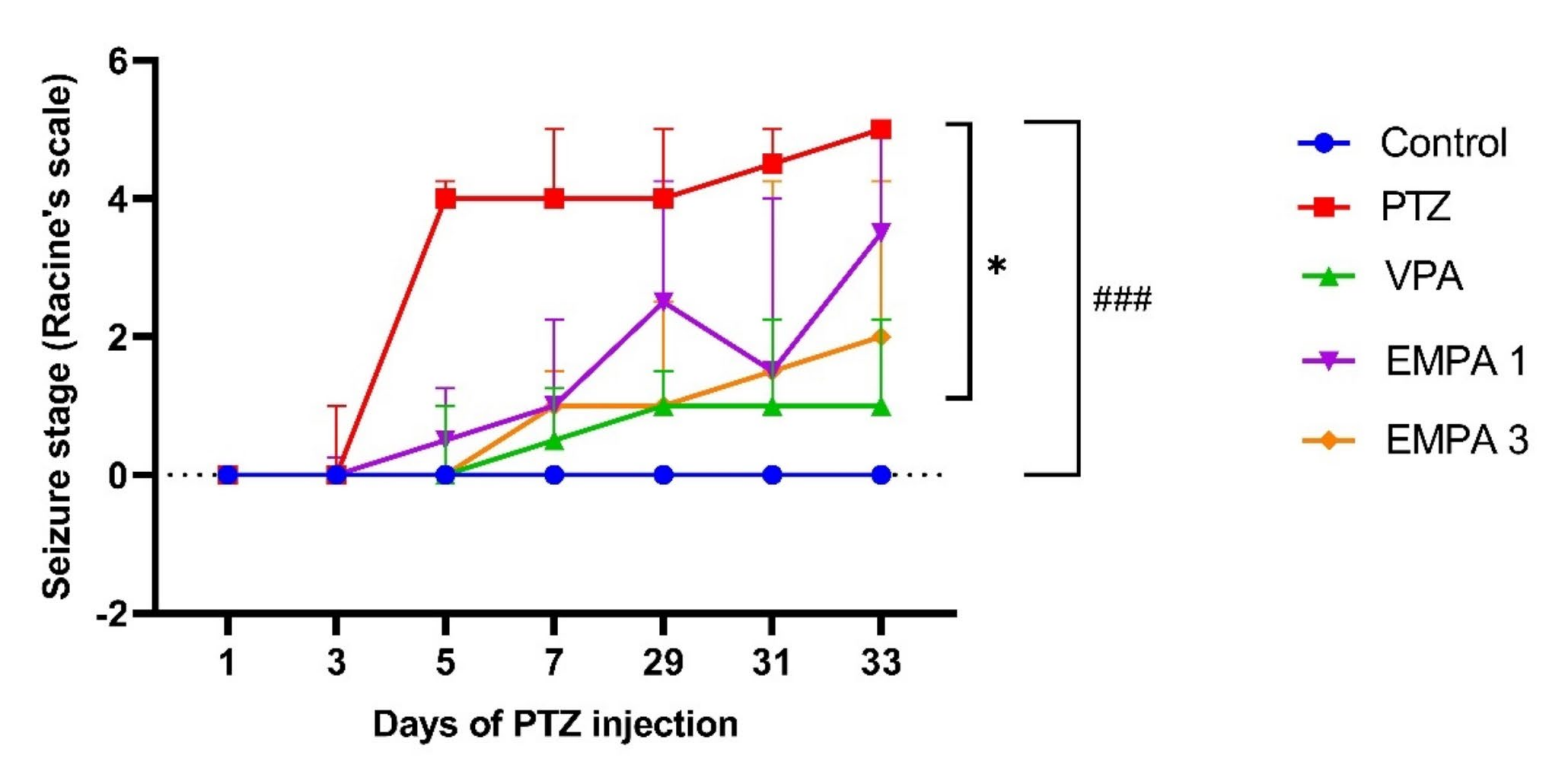
Effect of pretreatment with VPA and EMPA (1&3 mg/kg) on seizure severity in win- PTZ induced kindling epilepsy in rats according to Racine’s scale. Data are presented as median ± IQR, *n* = 6. Statistically significant differences are indicated as: ^####^ *P* < 0.001 versus control; *P < 0.05 versus PTZ using Friedman test followed by Dunn’s *post hoc* test. Racine’s scale: 0: for no response; 1: for facial and ear twitches; 2: for myoclonic jerks; 3: for myoclonic jerks with rearing; 4: for clonic convulsions with turning over into side position; 5: generalized tonic-clonic seizures or death. * PTZ was intraperitoneally injected (37.5 mg/kg) where rats received only seven injections: 4 initial doses day after day followed by 3 consecutive injections at the 29^th^, 31^st^, and 33^rd^ days to induce seizures in rats. VPA pretreatment was given at 200 mg/kg i.p. daily. EMPA pretreatments were given at 1 and 3 mg/kg orally daily. Abbreviations: PTZ: pentylenetetrazole, VPA: valproate, EMPA: empagliflozin, win-: window

The results indicated that PTZ kindling increased seizure severity significantly (*P* < 0.001) when compared to the control group. By the end of the experiment, the PTZ-kindled group was fully kindled with a seizure score of 4–5. The VPA group showed a significantly lower seizure score (*P* < 0.05) when compared to the PTZ group. However, the pretreatment with EMPA didn’t significantly affect the seizure score; the EMPA groups exhibited fewer convulsions with fewer facial twitches and myoclonic jerks and even didn’t show any generalized tonic clonic convulsions or death. No significant difference was observed between EMPA doses (Fig. 2).

**Fig. 3:**
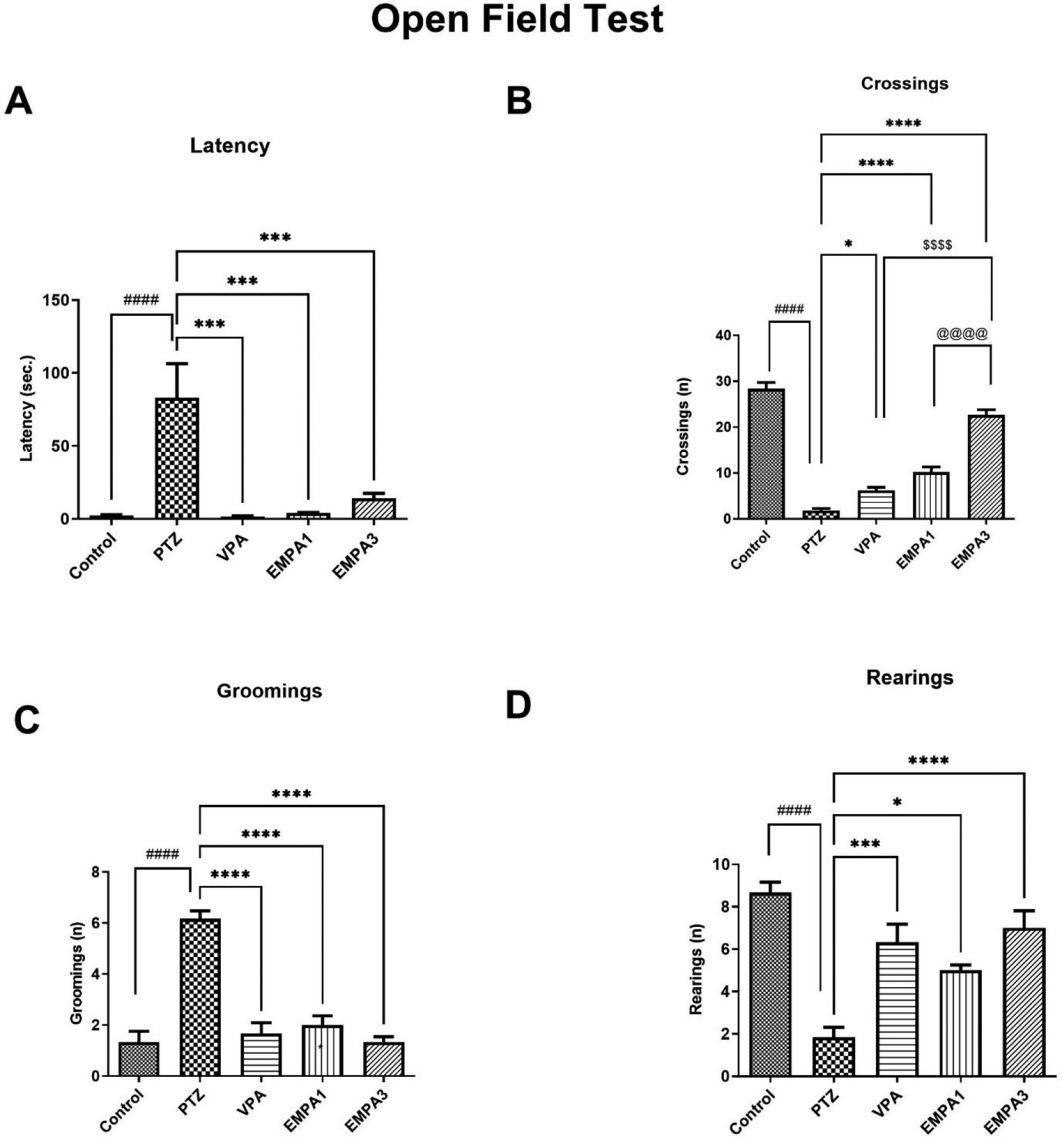
Effect of pretreatment with VPA and EMPA (1&3 mg/kg) on (**A**) latency, (**B**) crossings, (**C**) groomings and (**D**) rearings in open field test in win- PTZ induced kindling in rats. Bars are expressed as mean ± SEM, *n* = 6. Statistically significant differences are indicated as: ^####^ *P* < 0.0001 versus control; *P < 0.05, *** *P* < 0.001 and **** *P* < 0.0001 versus PTZ; ^$$$$^ *P* < 0.0001 versus VPA; ^@@@@^*P* < 0.0001 versus EMPA 3 using one-way ANOVA followed by *post-test* Tukey-Kramer. * PTZ was intraperitoneally injected (37.5 mg/kg) where rats received only seven injections: 4 initial doses day after day followed by 3 consecutive injections at the 29^th^, 31^st^, and 33^rd^ days to induce seizures in rats. VPA pretreatment was given at 200 mg/kg i.p. daily. EMPA pretreatments were given at 1 and 3 mg/kg orally daily. Abbreviations: PTZ: pentylenetetrazole, VPA: valproate, EMPA: empagliflozin, win-: window

Moreover, the impairments induced by PTZ in movement and memory were remarkably improved in the EMPA groups. EMPA decreased the time needed to move significantly (*P* < 0.001) (Fig. 3a), whereas it increased the number of crossings (squares) significantly (*P* < 0.0001) in the open field (Fig. 3b). Also, the increase in grooming behavior in the PTZ group was greatly countered in the EMPA groups (*P* < 0.0001) (Fig. 3c). Besides, motor coordination was greatly enhanced, as indicated by the significant increase in the number of rearings in the EMPA 1 (*P* < 0.05) and EMPA 3 (*P* < 0.0001) groups (Fig. 3d). As well, the VPA group showed significant improvements in a similar manner to EMPA in latency (*P* < 0.001), crossings (*P* < 0.05), grooming behavior (*P* < 0.0001), and rearing acts (*P* < 0.001) when compared to the PTZ group. The EMPA high dose (3 mg/kg) showed a significant and more pronounced improvement in the number of crossings in comparison to VPA (*P* < 0.0001), and the EMPA low dose (1 mg/kg) (*P* < 0.0001) (Fig. 3b).

### Effect of Pretreatment with VPA and EMPA (1&3 mg/kg) on Histopathological Changes in the Hippocampal Tissues (CA3) in win- PTZ Induced Kindling in rats: (I) Microscopic Pictures of H&E-stained Hippocampus Picked by the Light Microscope, and (II) Transmission Electron Micrographs of Hippocampal Cells

**Fig. 4:**
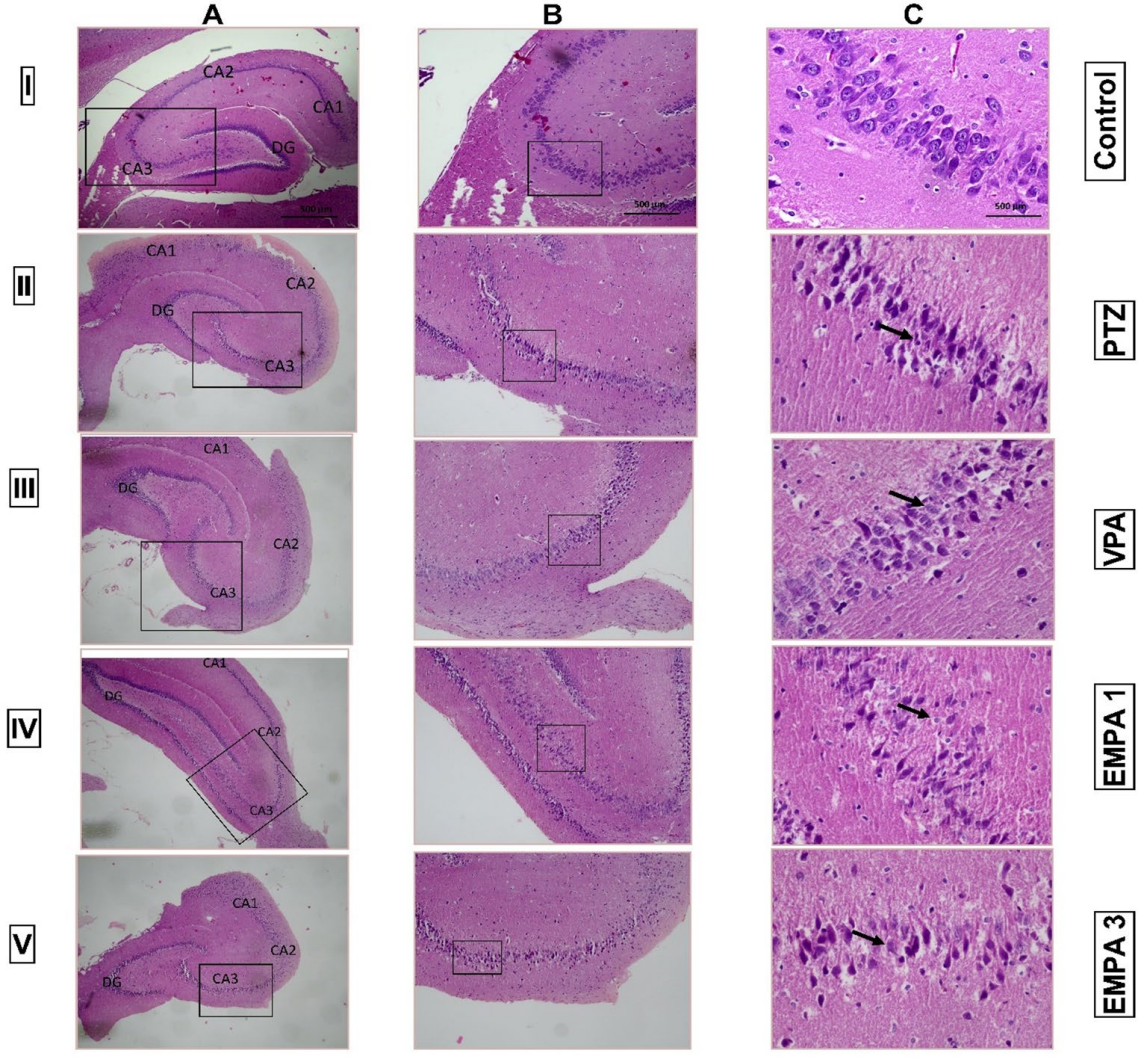
Effect of pretreatment with VPA and EMPA (1&3 mg/kg) on histopathological changes in the hippocampal region (CA3) in win- PTZ induced kindling in rats. Microscopic pictures of H&E-stained hippocampus picked up at (**A**) ×40 showing hippocampus proper CA1 to CA3 and DG subregions, and at (**B** & **C**) ×100 & ×400 respectively showing CA3 hippocampal region. **Control group**: showed normal pyramidal neurons in CA3 with preserved thickness (Figure IC, x400). **PTZ group**: showed diffuse loss of pyramidal neurons and a marked decrease in the thickness of CA3, oligodendroglia was seen in close position for the cell body of the degenerated neurons (satellitosis) (black-headed arrow) (Figure IIC, x400). **VPA group**: showed normal neurons with normal vacuolated nucleus and normal nucleoli (black-headed arrow), and the pyramidal neurons appeared with intensely basophilic cytoplasm (Figure IIIC, x400). **EMPA 1 group**: showed a slight loss of normal pyramidal neurons in CA3, and noticeable astrocytosis (black-headed arrow) (Figure IVC, x400). **EMPA 3 group**: showed normal neurons (black-headed arrow) and retained normal thickness of CA3 (Figure IVC, x400). * PTZ was intraperitoneally injected (37.5 mg/kg) where rats received only seven injections: 4 initial doses day after day followed by 3 consecutive injections at the 29^th^, 31^st^, and 33^rd^ days to induce seizures in rats. VPA pretreatment was given at 200 mg/kg i.p. daily. EMPA pretreatments were given at 1 and 3 mg/kg orally daily. Abbreviations: PTZ: pentylenetetrazole, VPA: valproate, EMPA: empagliflozin, win-: window, H&E: hematoxylin and eosin, CA1: Cornu Ammonis 1, CA3: Cornu Ammonis 3, DG: dentate gyrus

The normal architecture of the cornu ammonis 3 (CA3) hippocampal region is characterized by a pyramidal cell layer. However, the PTZ-challenged group showed degenerated neurons with noticeable loss of the pyramidal layer of the CA3 region. Satellitosis was also observed (Fig. 4, IIc). In contrast, VPA showed improvements in both the integrity and the number of neurons in the CA3 region, and the microscopic pictures demonstrated normal neurons in the pyramidal layer with intensely basophilic cytoplasm (Fig. 4, IIIC). Moreover, EMPA 1 could retain normal pyramidal neurons in spite of showing slight neuronal loss and astrocytosis (Fig. 4, IVC). Interestingly, the EMPA 3 group could nearly maintain the normal thickness of the CA3 area (Fig. 4., VC).

**Fig. 5:**
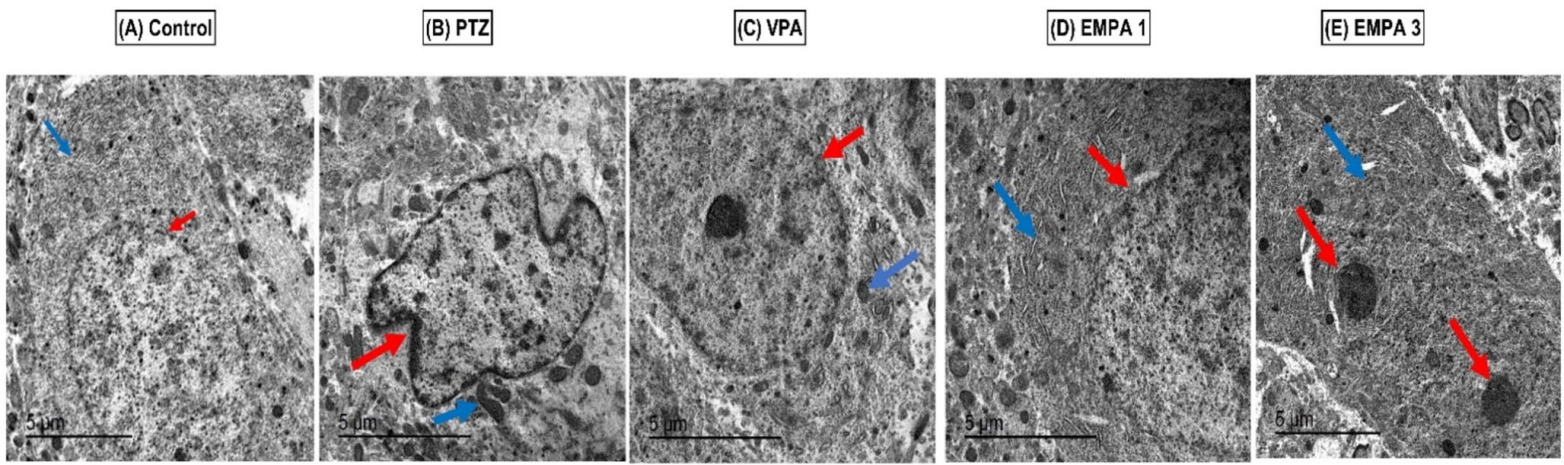
Transmission electron micrographs of the hippocampal cells showing the effect of pretreatment with VPA and EMPA (1&3 mg/kg) on the hippocampal cell structure in win- PTZ induced kindling in rats. **Control group** (Fig. 5A): The neuron is displaying a normal nucleus with normal euchromatin and a normal nuclear membrane (red arrow), with normal rough endoplasmic reticulum (blue arrow) and normal mitochondria in the cytoplasm. **PTZ group** (Fig. 5B): The neuron showed shrinkage of the nuclear membrane (red arrow) and increased heterochromatin with marked loss of the rough reticulum and C-shaped mitochondria in the cytoplasm (blue arrow). **VPA group** (Fig. 5C): The neuron is displaying a normal nucleus with a normal euchromatin, nucleoli, and normal nuclear membrane (red arrow), with normal rough endoplasmic reticulum and normal mitochondria (blue arrow) in the cytoplasm. **EMPA 1** (Fig. 5D): The neuron is displaying irregularity and dentation of the nuclear membrane (red arrow), with a normal rough endoplasmic reticulum in the cytoplasm (blue arrow). **EMPA 3** (Fig. 5E): The neuron is displaying electron-dense apoptotic bodies in the cytoplasm (red arrow); however, it showed a normal rough endoplasmic reticulum (blue arrow). * PTZ was intraperitoneally injected (37.5 mg/kg) where rats received only seven injections: 4 initial doses day after day followed by 3 consecutive injections at the 29^th^, 31^st^, and 33^rd^ days to induce seizures in rats. VPA pretreatment was given at 200 mg/kg i.p. daily. EMPA pretreatments were given at 1 and 3 mg/kg orally daily. Abbreviations: PTZ: pentylenetetrazole, VPA: valproate, EMPA: empagliflozin, win-: window

Electron micrographs of the hippocampal cells of the control group displayed a normal nucleus with normal euchromatin, a normal nuclear membrane, a rough endoplasmic reticulum, and mitochondria in the cytoplasm. However, PTZ electron micrographs demonstrated a shrunken nuclear membrane and increased heterochromatin, with marked loss of the rough endoplasmic reticulum and disrupted mitochondria (C-shaped) in the cytoplasm. The VPA group revealed marked improvement in the cell components, with morphological similarities to the normal control group. Meanwhile, treatment with both EMPA (1 mg/kg) and EMPA (3 mg/kg) could maintain the normal rough endoplasmic reticulum in the cytoplasm (Fig. 5).

### Effect of Pretreatment with VPA and EMPA (1&3 mg/kg) on GSH and MDA Contents in Hippocampal Sections in win- PTZ Induced Kindling in rats

**Fig. 6:**
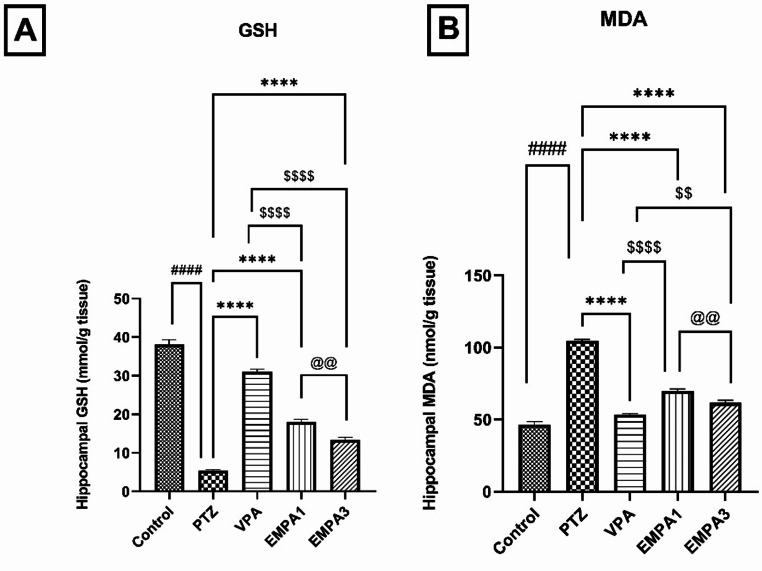
Effect of pretreatment with VPA and EMPA (1&3 mg/kg) on (**A**) antioxidant activity of non-enzymatic (GSH) and (**B**) malondialdehyde (MDA) contents in hippocampal sections in win- PTZ induced kindling in rats. Bars are expressed as mean ± SEM, *n* = 6. Statistically significant differences are indicated as: ^####^ *P* < 0.0001 versus control; **** *P* < 0.0001 versus PTZ; ^$$^ *P* < 0.01 and ^$$$$^ *P* < 0.0001 versus VPA; and ^@@^*P* < 0.01 versus EMPA 3 using one-way ANOVA followed by *post-test* Tukey-Kramer. * PTZ was intraperitoneally injected (37.5 mg/kg) where rats received only seven injections: 4 initial doses day after day followed by 3 consecutive injections at the 29^th^, 31^st^, and 33^rd^ days to induce seizures in rats. VPA pretreatment was given at 200 mg/kg i.p. daily. EMPA pretreatments were given at 1 and 3 mg/kg orally daily. Abbreviations: PTZ: pentylenetetrazole, VPA: valproate, EMPA: empagliflozin, win-: window, GSH: reduced glutathione, MDA: malondialdehyde

PTZ-kindling increased the oxidative stress burden, as indicated by the significant reduction (*P* < 0.0001) in the GSH and the significant enhancement (*P* < 0.0001) of MDA hippocampal contents when compared to the control group. Pretreatment with EMPA (1 & 3 mg/kg) improved the oxidative stress status (*P* < 0.0001) and decreased the tissue damage induced by PTZ. That was indicated by the significant enhancement of GSH (*P* < 0.0001) and reduction of MDA contents (*P* < 0.0001), respectively. Despite EMPA 1 showing a significant increase in GSH contents (*P* < 0.01) relative to EMPA 3, EMPA 3 attenuated the rise in lipid peroxidation (MDA levels) induced by PTZ more significantly (*P* < 0.01) than EMPA 1. Meanwhile, the VPA group exhibited better results than the EMPA groups significantly in both GSH (*P* < 0.0001) and MDA contents (*P* < 0.0001; compared to EMPA 1) and (*P* < 0.01; compared to EMPA 3). (Figure 6a and b).

### Effect of Pretreatment with VPA and EMPA (1&3 mg/kg) on BDNF and CREB Contents in Hippocampal Sections in win- PTZ Induced Kindling in rats

**Fig. 7:**
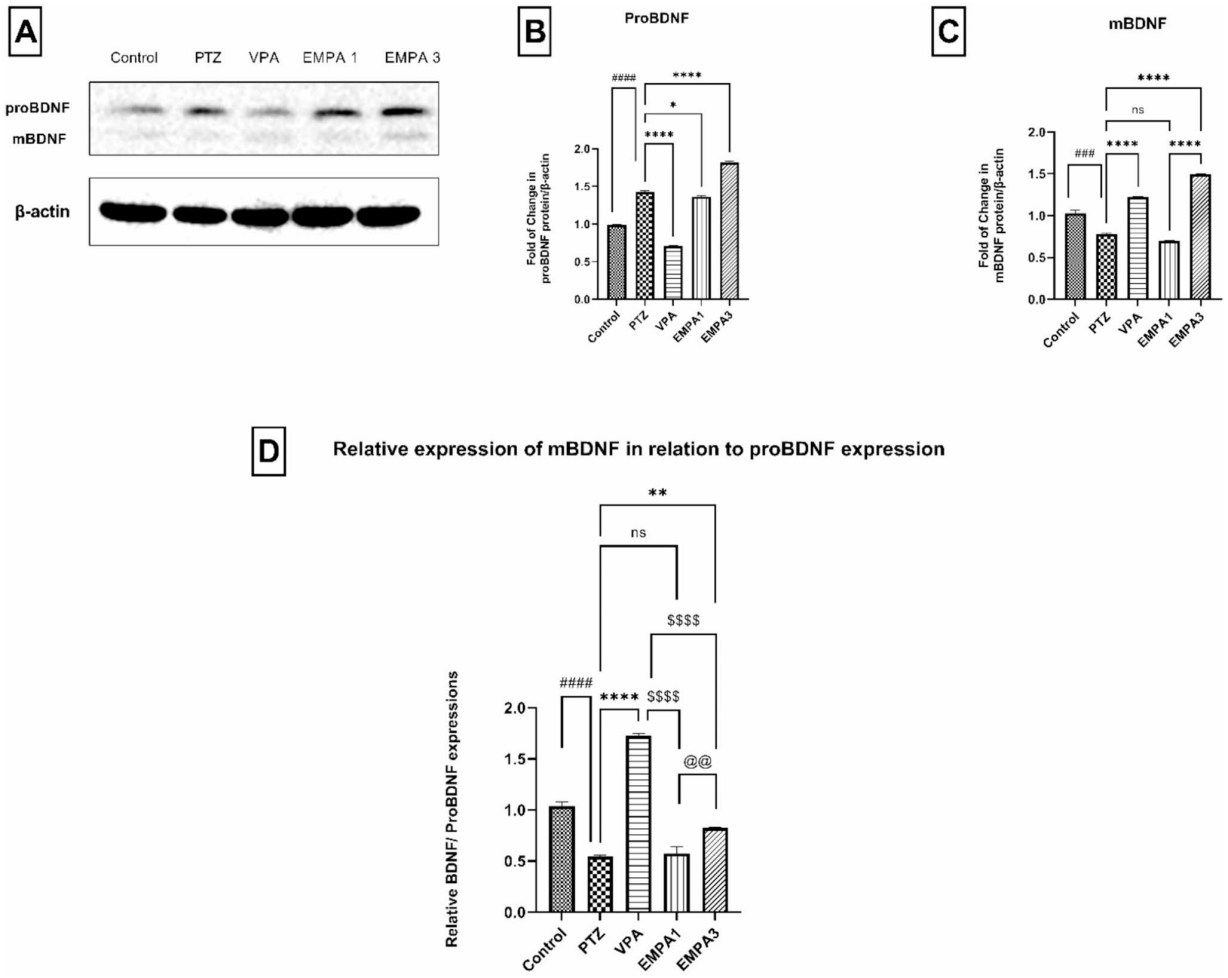
Effect of pretreatment with VPA and EMPA (1&3 mg/kg) on proBDNF, mBDNF, and relative mBDNF in relation to proBDNF levels in hippocampal sections in win- PTZ induced kindling in rats. **A**) Western blot analysis of BDNF. **B**) Fold of change of proBDNF protein/ β-Actin. **C**) Fold of change of mBDNF protein/ β-Actin. D) Relative mBDNF/proBDNF expression. Data are expressed as mean ± SEM, *n* = 3. Statistically significant differences are indicated as: ^####^ *P* < 0.0001 versus control; * *P* < 0.05, ** *P* < 0.01, and **** *P* < 0.0001 versus PTZ; ^$$$$^ *P* < 0.0001 versus VPA; and ^@@^ *P* < 0.01 versus EMPA 3 using one-way ANOVA followed by *post-test* Tukey-Kramer. * PTZ was intraperitoneally injected (37.5 mg/kg) where rats received only seven injections: 4 initial doses day after day followed by 3 consecutive injections at the 29^th^, 31^st^, and 33^rd^ days to induce seizures in rats. VPA pretreatment was given at 200 mg/kg i.p. daily. EMPA pretreatments were given at 1 and 3 mg/kg orally daily. Abbreviations: PTZ: pentylenetetrazole, VPA: valproate, EMPA: empagliflozin, win-: window, proBDNF: pro brain-derived neurotrophic factor, mBDNF: mature brain-derived neurotrophic factor

Western blot analysis (Fig. 7a) reflected that PTZ induced proBDNF release, as indicated by the significant increase (*P* < 0.0001) in proBDNF contents relative to the control group (Fig. 7b). On the other hand, the relative expression of mBDNF to proBDNF was significantly decreased (*P* < 0.0001) (Fig. 7d). Both VPA and EMPA 3 showed marked activation of the precursor form of BDNF (proBDNF) to the mature form (mBDNF), as indicated by the significant relative elevation of mBDNF contents when compared to the PTZ group (*p* < 0.0001 and *p* < 0.01, respectively). Also, the relative mBDNF to proBDNF expressions of VPA and EMPA3 were significantly higher (*p* < 0.0001 and *p* < 0.01, respectively) when compared to EMPA 1 (Fig. 7d). Notably, VPA encouraged significant activation (*P* < 0.0001) of the proBDNF precursor when compared to EMPA 3. In addition, the EMPA 1 group showed sufficient formation of the mBDNF isoform but wasn’t significantly different from the PTZ group (Fig. 7d).

**Fig. 8:**
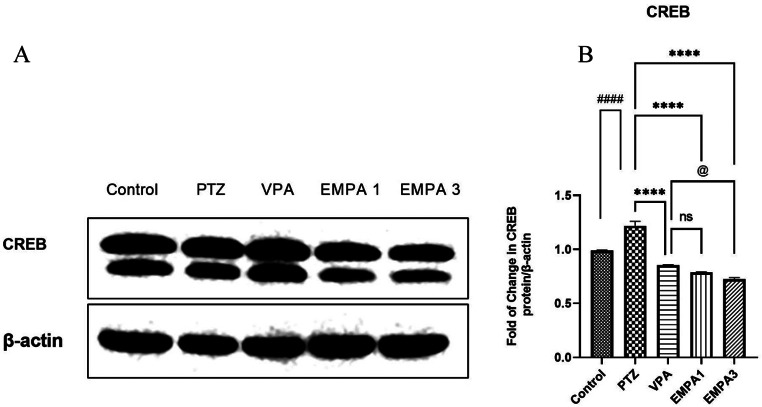
Effect of pretreatment with VPA and EMPA (1&3 mg/kg) on CREB protein levels in hippocampal sections in win- PTZ induced kindling in rats. **A**) Western blot analysis of CREB. **B**) Fold of change of CREB protein/ β-Actin. Data are expressed as mean ± SEM, *n* = 3. Statistically significant differences are indicated as: ^####^ *P* < 0.0001 versus control; **** *P* < 0.0001 versus PTZ; and ^@^ *P* < 0.05 versus EMPA 3 using one-way ANOVA followed by *post-test* Tukey-Kramer. * PTZ was intraperitoneally injected (37.5 mg/kg) where rats received only seven injections: 4 initial doses day after day followed by 3 consecutive injections at the 29^th^, 31^st^, and 33^rd^ days to induce seizures in rats. VPA pretreatment was given at 200 mg/kg i.p. daily. EMPA pretreatments were given at 1 and 3 mg/kg orally daily. Abbreviations: PTZ: pentylenetetrazole, VPA: valproate, EMPA: empagliflozin, win-: window, CREB: cyclic adenosine monophosphate (cAMP) response element binding protein

Analysis of CREB expression (43 kDa) by western blotting (Fig. 8a) revealed that PTZ led to a significant elevation (*P* < 0.0001) of CREB contents when compared to the control group (Fig. 8b). This elevation was significantly ameliorated in the treatment groups, where VPA, EMPA 1, and EMPA 3 showed significantly reduced levels of CREB (*p* < 0.0001) (Fig. 8b). CREB levels were significantly lower in the EMPA 3 group compared to the VPA group (*P* < 0.05).

### Effect of Pretreatment with VPA and EMPA (1&3 mg/kg) on Immune-Expression of Apoptotic Marker (BAX) and Astrocyte Proliferation Marker (GFAP) in Hippocampal Sections in win- PTZ Induced Kindling in rats

**Fig. 9:**
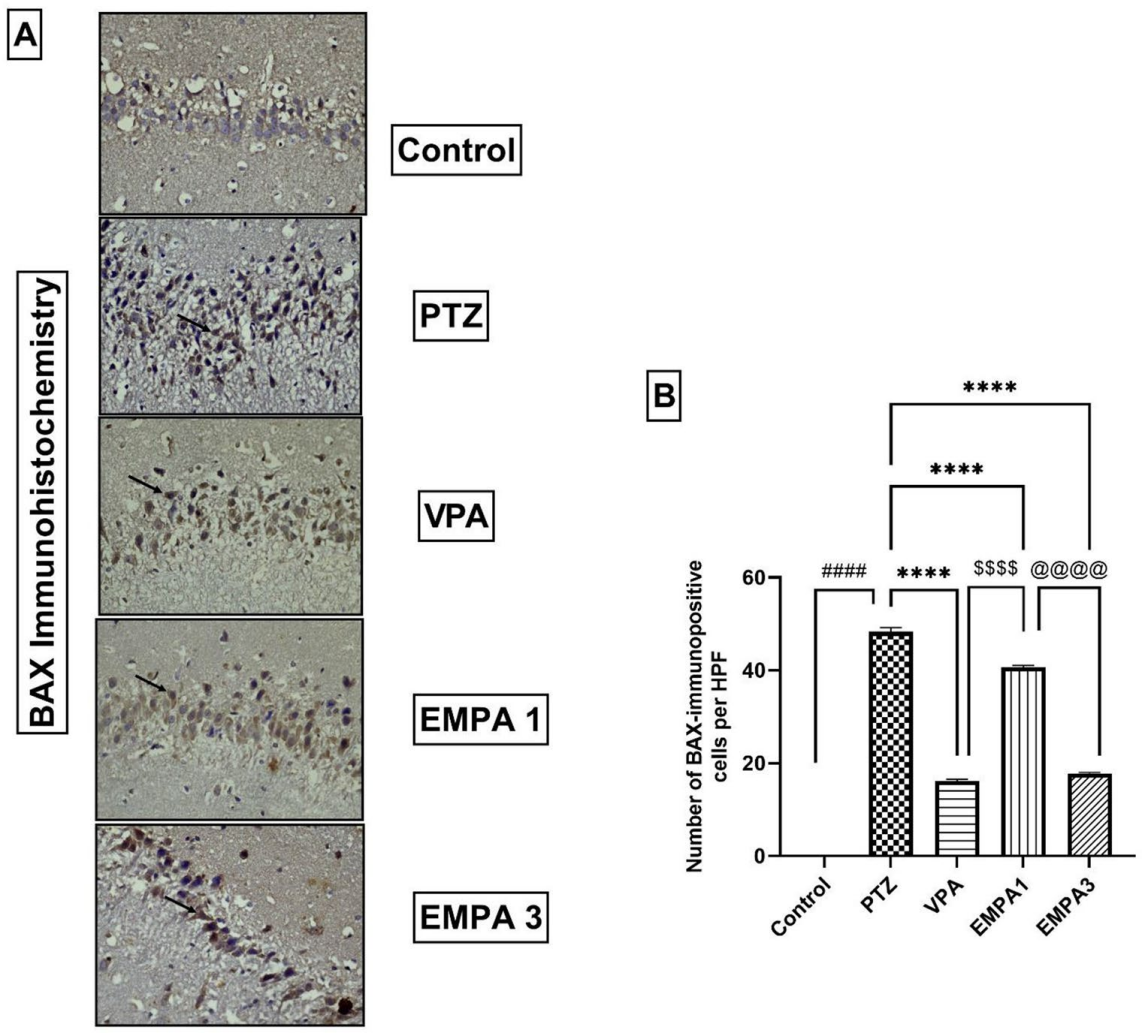
Effect of pretreatment with VPA and EMPA (1&3 mg/kg) on (**A**) immune-expression of apoptotic marker (BAX) and (**B**) Quantification of BAX-immunopositive cells in hippocampal sections in win-PTZ induced kindling in rats. Data are expressed as mean ± SEM, *n* = 6. Statistically significant differences are indicated as: ^####^ *P* < 0.0001 versus control; **** *P* < 0.0001 versus PTZ; ^$$$$^ *P* < 0.0001 versus VPA; and ^@@@@^ *P* < 0.0001 versus EMPA 3 using one-way ANOVA followed by *post-test* Tukey-Kramer. Figure 9A (Immunostaining against BAX, hematoxylin as a counterstain, x400) **Control group**: CA3 is displaying negative immunostaining against BAX. **PTZ group**: CA3 is displaying diffuse brown immunostaining in the cytoplasm of the neurons against BAX. **VPA group**: CA3 is displaying mild immunopositive brown staining in the cytoplasm of the neurons against BAX. **EMPA 1**: CA3 is displaying moderate immunopositive brown staining in the cytoplasm of the neurons against BAX. **EMPA 3**: CA3 is displaying mild immunopositive brown staining in the cytoplasm of the neurons against BAX (see arrows). * PTZ was intraperitoneally injected (37.5 mg/kg) where rats received only seven injections: 4 initial doses day after day followed by 3 consecutive injections at the 29^th^, 31^st^, and 33^rd^ days to induce seizures in rats. VPA pretreatment was given at 200 mg/kg i.p. daily. EMPA pretreatments were given at 1 and 3 mg/kg orally daily. Abbreviations: PTZ: pentylenetetrazole, VPA: valproate, EMPA: empagliflozin, win-: window, BAX: BCL2-Associated X Protein

Results demonstrated that PTZ induced apoptosis in the hippocampal tissues, where the PTZ group showed diffuse brown immunostaining against BAX in the cytoplasm of CA3 neurons (Fig. 9a). Treatments (VPA and EMPA) ameliorated apoptosis by expressing less immunopositive brown staining against BAX in the cytoplasm of CA3 neurons compared to the PTZ group. The number of BAX-positive immune cells was conducted quantitatively, and results confirmed our observations as VPA and EMPA 3 showed a reduced number of BAX-positive immune cells significantly when compared to the PTZ group (*P* < 0.0001). Furthermore, compared to the EMPA 1 group, the EMPA 3 group reduced apoptosis more significantly (*P* < 0.0001) (Fig. 9b).

**Fig. 10:**
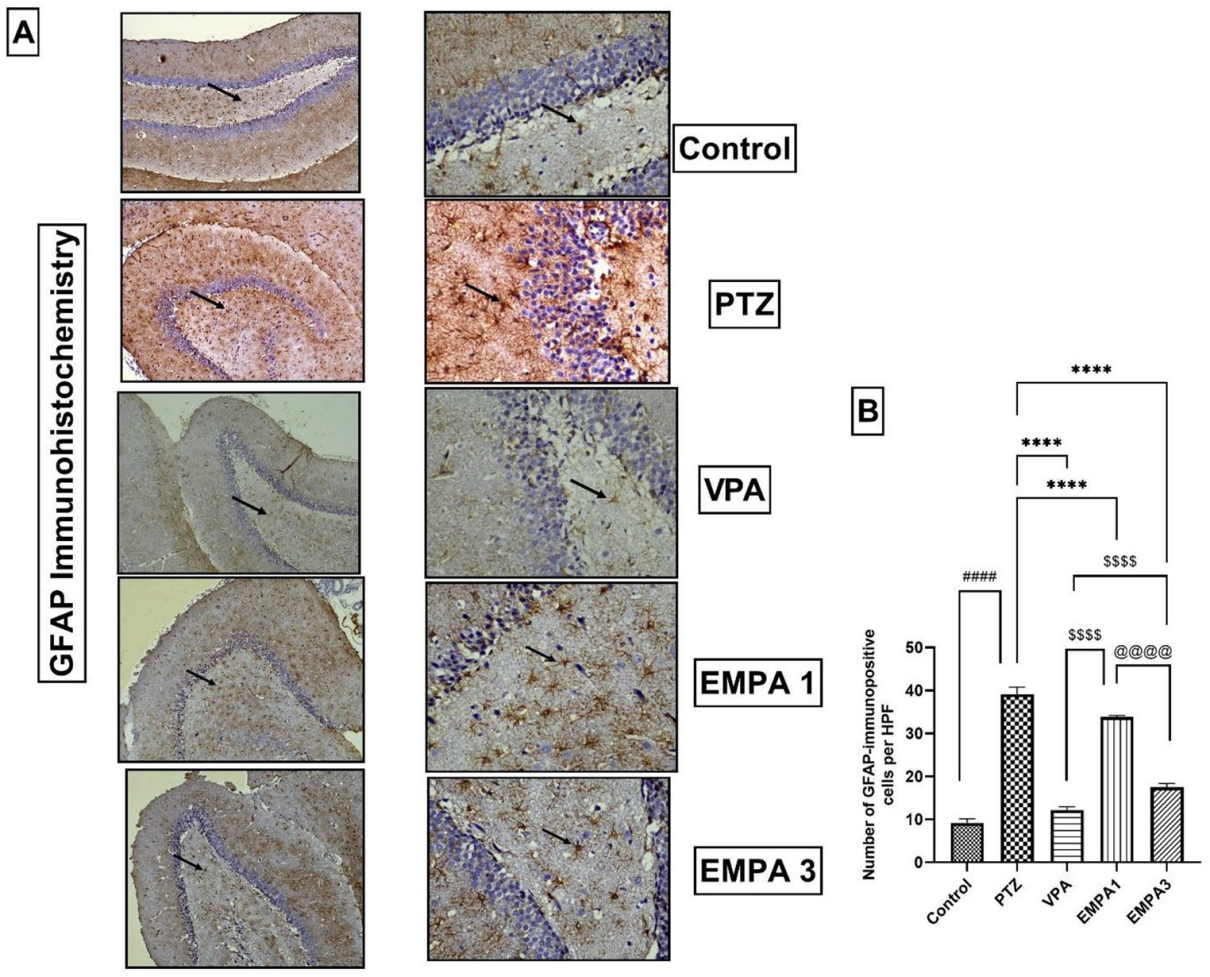
Effect of pretreatment with VPA and EMPA (1&3 mg/kg) on immune-expression of GFAP marker (**A**: x100, **B**: x400), and **C**) Quantification of GFAP-immunopositive cells in hippocampal sections in win- PTZ induced kindling in rats. Data are expressed as mean ± SEM, *n* = 6. Statistically significant differences are indicated as: ^####^ *P* < 0.0001 versus control; **** *P* < 0.0001 versus PTZ; ^$$$$^ *P* < 0.0001 versus VPA; and ^@@@@^ *P* < 0.0001 versus EMPA 3 using one-way ANOVA followed by *post-test* Tukey-Kramer. Figure 10A: (Immunostaining against GFAP, hematoxylin as counter stain, x100 (**A**) & x400 (**B**)) **Control group**: Dentate gyrus showed normal astrocytes. **PTZ group**: Marked astrogliosis, reactive astrocytosis, (alteration of astrocytes and increase in their number) in dentate gyrus. **VPA group**: normal astrocyte in the area of dentate gyrus. **EMPA 1**: showed moderate astrogliosis in the dentate gyrus. **EMPA 3**: showed mild astrogliosis in the dentate gyrus (see arrows). * PTZ was intraperitoneally injected (37.5 mg/kg) where rats received only seven injections: 4 initial doses day after day followed by 3 consecutive injections at the 29^th^, 31^st^, and 33^rd^ days to induce seizures in rats. VPA pretreatment was given at 200 mg/kg i.p. daily. EMPA pretreatments were given at 1 and 3 mg/kg orally daily. Abbreviations: PTZ: pentylenetetrazole, VPA: valproate, EMPA: empagliflozin, win-: window, GFAP: glial fibrillary acidic protein

The figures showed normal astrocytes in the area of the dentate gyrus of hippocampal tissues in the control group (Fig. 10a). However, the PTZ group exhibited marked astrocytosis and astrogliosis (Fig. 10a), which was confirmed by the statistical upregulation of the GFAP-positive immune cells (*P* < 0.0001) (Fig. 10b). VPA and EMPA pretreatments showed significantly reduced GFAP immunoreactivity in the hippocampal tissues of the PTZ-kindled rats (*P* < 0.0001). However, the VPA group showed significantly reduced expression of GFAP levels when compared to the EMPA 3 group (*P* < 0.0001). Moreover, EMPA treatment mitigated reactive astrocytosis in a dose-dependent manner; GFAP expression was significantly reduced in EMPA 3 (*P* < 0.0001) compared to EMPA 1 (Fig. 10b).

### Effect of Pretreatment with VPA and EMPA (1&3 mg/kg) on Hippocampal Contents of Npas4 in) PTZ Induced Kindling in rats

**Fig. 11:**
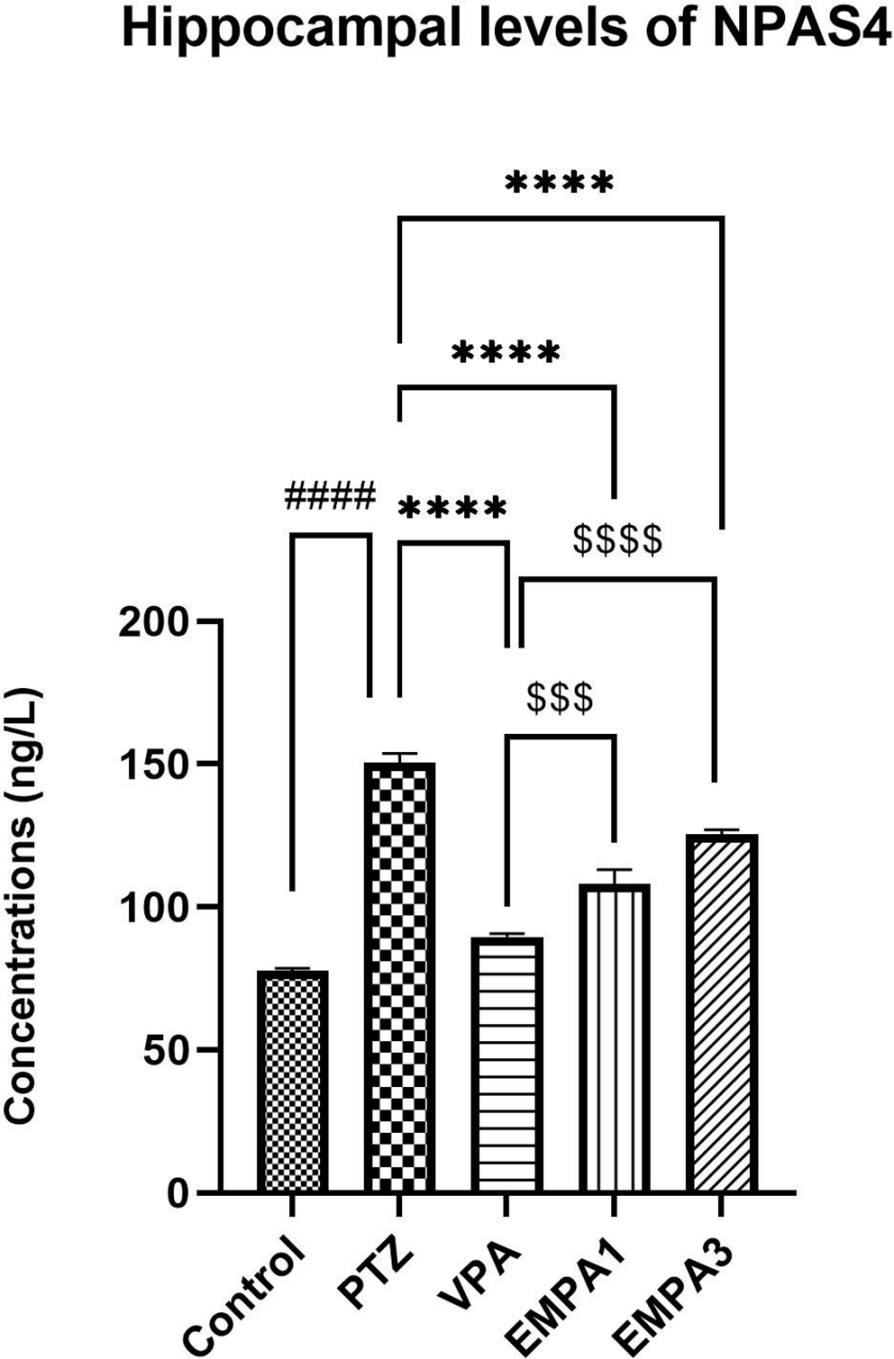
Effect of pretreatment with VPA and EMPA (1&3 mg/kg) on hippocampal contents of Npas4 in win- PTZ induced kindling in rats. Bars are expressed as mean ± SEM, *n* = 6. Statistically significant differences are indicated as: ^####^ *P* < 0.0001 versus control; **** *P* < 0.0001 versus PTZ; ^$$$^ *P* < 0.001 and ^$$$$^ *P* < 0.0001 versus VPA using one-way ANOVA followed by *post-test* Tukey-Kramer. * PTZ was intraperitoneally injected (37.5 mg/kg) where rats received only seven injections: 4 initial doses day after day followed by 3 consecutive injections at the 29^th^, 31^st^, and 33^rd^ days to induce seizures in rats. VPA pretreatment was given at 200 mg/kg i.p. daily. EMPA pretreatments were given at 1 and 3 mg/kg orally daily. Abbreviations: PTZ: pentylenetetrazole, VPA: valproate, EMPA: empagliflozin, win-: window, NPAS4: neuronal PAS domain Protein 4

Results showed a significant increase in the hippocampal contents of Npas4 induced by PTZ relative to the control group (*P* < 0.0001) (Fig. 11). However, both VPA and EMPA groups markedly reduced Npas4 expression in relation to the PTZ group (*P* < 0.0001) (Fig. 11). Compared to the EMPA 1 (*P* < 0.001) and EMPA 3 (*P* < 0.0001) groups, VPA could significantly lower Npas4 contents while maintaining concentrations close to those of the control group.

## Discussion

In this study, we tried to give rise to a new therapeutic agent for epilepsy and attract interest in studying new mechanisms of EMPA other than the known ones. The treatment strategy based on the BDNF-TrkB pathway, which is thought to be implicated in the epileptogenesis process, was investigated. EMPA significantly improved the motor dysfunction and cognitive impairment induced by PTZ, as demonstrated by seizure scoring and behavioral testing. These findings were confirmed by the histopathological examination, which elucidated the effect of EMPA on restoring cell integrity and alleviating neuronal damage in the hippocampal areas after PTZ administration. Interestingly, EMPA was found to exert potential protective effects through the marked reduction of hippocampal oxidative stress and tissue damage induced by the chronic administration of PTZ, as evidenced by significantly increasing GSH and decreasing MDA hippocampal contents. In addition, pretreatment with EMPA groups impaired the hippocampal apoptotic pathways triggered by chronic PTZ-induced seizures. That was indicated by significantly reduced BAX expression in the EMPA group compared to the PTZ group. In addition, the expression of GFAP was significantly reduced. Accordingly, astrogliosis was remarkably impaired in EMPA groups, indicating reduced brain injury and neuroprotection. Moreover, the findings suggested the implication of EMPA in resetting the balance between excitatory and inhibitory circuits. That was indicated by enhancing the activity of the active isomer, mBDNF, that controls the inhibitory synapses and promotes cell survival and neurogenesis. Besides, EMPA attenuated the excitatory inputs induced by PTZ through significant inhibition of Npas4 expression and CREB upregulation. These promising results were more pronounced at the high dose of EMPA (3 mg/kg) than at the lower dose (1 mg/kg), which indicated that EMPA may exert its effects in a dose-dependent manner.

SGLT2 inhibitors are one of the newest classes of drugs that have been approved for treatment of type 2 diabetes (Hsia et al. 2017). SGLTs were firstly merely considered as Na^+^/glucose transporters. Yet, recent studies revealed novel and physiological roles of SGLT2 for being widely distributed in brain, especially glutamatergic pyramidal cells in the CA1 region of the hippocampus, and other organs rather than the intestine and kidney. Interestingly, SGLTs were found to be involved in learning processes, appetite, energy and glucose homeostasis, and central cardiovascular and autonomic regulation (Pawlos et al. 2021). Moreover, cells expressing SGLTs became more likely known as glucose sensors, as they were thought to respond to extracellular glucose concentration affecting depolarization of membrane potentials (Wright et al. 2011); (Wright et al. 2017). Hence, rat hypothalamic glucose-excited neurons were blocked using SGLT inhibitor phloridzin (O’Malley et al. 2006). The lipid solubility and low molecular weights of SGLT2 inhibitors, in addition to the impaired integrity of blood brain barrier (BBB) in most endocrine and neurological disorders, support the notion that SGLT2 inhibitors may be able to penetrate BBB and exert their neuroprotective effects through SGLT2 that are highly expressed in the CNS (Pawlos et al. 2021). VPA is still one of the most widely used and effective antiepileptic drugs for most types of epilepsy (Campos et al. 2018), so it was useful to consider VPA treatment in this study as a reference to compare the relative proposed antiepileptic effect of EMPA to it.

Epileptogenesis is defined as the latent period between the initial brain insult and the first unprovoked seizure, where very intensive neuronal alterations take place, including oxidative stress, the expression of pro-inflammatory mediators, neurodegeneration, reactive gliosis, damage to the blood-brain barrier, increased epileptiform spiking, and spontaneous recurrent seizures (Borowicz-Reutt and Czuczwar 2020). PTZ is a gamma-aminobutyric acid (GABA)-A receptor antagonist, where GABA is the main inhibitory neurotransmitter in the mammalian brain (Thapliyal and Babu 2018). In the kindling model, chronic administration of subconvulsive doses of PTZ initiates the recurrence of seizures in a similar manner to the human epilepsy more than the acute models, where the seizure severity increases in a stepwise manner with each PTZ injection inducing severe generalized tonic-clonic seizures (Shimada and Yamagata 2018). In the current study, the win-kindling protocol involved PTZ injections with a free-injection time period between the initial and final injections to allow a set of neural changes that increase the susceptibility of the brain to seizures, as stated by Davoudi et al. (Davoudi et al. 2013), and to greatly mimic the epileptogenesis process. Interestingly, the present study showed that the repetitive injection of PTZ increased the severity of seizures significantly, with the generation of generalized tonic-clonic convulsions, death, and a fully-kindled state. EMPA attenuated the seizure severity but not significantly; however, the number of fully-kindled rats was lower. In addition, EMPA showed an anticonvulsant effect by preventing generalized tonic-clonic convulsions or death.

A previous study reported that anxiety accelerates the development of kindling and that PTZ-induced kindling is associated with an increase in anxiety and emotional tension characterized by behavioral and locomotor deficits in the open field test (Godlevsky et al. 2014). In line with this study, PTZ significantly decreases the tendency to move and explore by increasing latency and decreasing the number of crossings and rearing acts in the open field. In addition, grooming, as a state of stress (Katz and Roth 1979), was increased. However, a significant improvement in motor functions and behavior responses in EMPA-treated groups was observed. Moreover, EMPA 3 outperformed EMPA 1 and even VPA in increasing the number of crossings. EMPA was previously proven to improve the ambulation rate and neurological functions in a dose-dependent manner in cerebral ischemia/reperfusion (I/R) in a rat model (Abdel-latif et al. 2020), which supported the current results.

The CA3 region has been recently studied as one of the most susceptible regions to seizures and neurotoxicity in the hippocampus (Cherubini and Miles 2015). The normal architecture of the CA3 hippocampal region is characterized by a pyramidal cell layer where neurons displayed rounded central vesicular nucleus and basophilic cytoplasm (Amin et al. 2020). PTZ kindling models exhibited neuronal damage characterized by marked cell loss and apoptosis in the hippocampal CA1 and CA3 layers, in addition to decrease in the thickness of the pyramidal cell layer (Gao et al. 2018). In the present study, the damage induced by PTZ in the hippocampal region was characterized by loss of pyramidal cell integrity and thickness and satellitosis, a hallmark of pathological conditions (Civita et al. 2020). Also, a shrunken nuclear membrane and increased heterochromatin, with marked loss of the rough endoplasmic reticulum and disrupted mitochondria, were observed in the electron micrographs. EMPA improved the histopathological abnormalities induced by PTZ, suggesting a neuroprotective effect; however, the improvement was more pronounced in 3 mg/kg EMPA through preserving the normal thickness of the pyramidal cell layer. This neuroprotective effect may influence the decrease in seizure severity as well as the behavior improvement indicated by the open field test. Meanwhile, VPA was superior in restoring the integrity and thickness of the pyramidal cells and neuron cell components, including the nucleus and mitochondria.

The excessive production of free radicals may initiate neuronal hyperexcitability (Geronzi et al. 2018). Oxidative stress is believed to be one of the factors that trigger epilepsy, as it’s involved in mitochondrial dysfunction, neuronal death, and seizures (Aguiar et al. 2012). This explains the relationship between oxidative stress and epileptogenesis as well as the role of antioxidant-based therapy in the management of epilepsy (Geronzi et al. 2018). GSH is one of the most important defense mechanisms against oxidative stress through scavenging hydroxyl radicals and singlet oxygen directly, in addition to the regeneration of important antioxidants such as vitamins E and C (Méndez-Armenta et al. 2014). MDA, as one of the mitochondrial lipid peroxidation products, has been implicated in mitochondrial oxidative stress in the hippocampus in different experimental models (Shin et al. 2008). Besides, blood samples from epileptic patients have shown low levels of antioxidants, which have been improved after treatment (Sudha et al. 2001). In the current study, EMPA could exert a neuroprotective potential against PTZ-induced seizures through significant enhancing the antioxidant capacity by increasing GSH activity and lowering MDA contents in the hippocampus after oxidative stress induced by PTZ. This supported the findings that EMPA could show cerebral benefits as antioxidant through modulation of the NF-κB/Nrf-2/PPAR-γ axis (Abdelhamid et al. 2020).

Experimental studies suggested that prolonged seizures induce mitochondrial dysfunction and oxidative stress, eventually leading to neuronal apoptosis (Méndez-Armenta et al. 2014). Seizures initiate the apoptotic pathway through depleting energy and the accumulation of Ca^+ 2^ inside the mitochondria, activating both extrinsic (expression of death receptors on the cell surface) and intrinsic factors (mitochondrial dysfunction) that in turn induce BAX release. Both BAX and mitochondrial calcium induce cytochrome c release from the mitochondria, which triggers the activation of both caspase 9 and caspase 3, respectively, leading to neuronal cell death (Niquet and Wasterlain 2004). EMPA, specifically, was proven to exert antiapoptotic effects through crossing the damaged BBB and repairing the neurovascular unit (NVU) structure (Dong et al. 2022). In addition, EMPA has demonstrated an antiapoptotic effect in brain tissues in experimental rats after stroke (Abdel-latif et al. 2020). This was in agreement with the current findings that indicated the antiapoptotic effects and the repairing capability of EMPA in the hippocampal regions, where EMPA showed significantly reduced immunoreactive pro-apoptotic BAX proteins in the hippocampal sections in a dose-dependent manner compared to the PTZ group.

Epileptogenesis propagates inflammatory pathways as well as the reactivity of glial cells, including astrocytes. Astrogliosis, or reactive astrocytosis, refers to morphological, biochemical, or even functional alterations in astrocytes that may take place as a defense mechanism after brain injury (Hayatdavoudi et al. 2022). These alterations include the upregulation of GFAP and cellular hypertrophy (Attia et al. 2019); hence, GFAP is considered a marker of astrocyte proliferation (astrocytosis) and reactive astrogliosis that take place under pathological conditions (Hubbard and Binder 2016).

Recent studies have stated that inflammatory cascades may play a role in astrogliosis by inhibiting glutamate uptake at astrocytic membranes (Bezzi et al. 2001); (Coulter and Steinhäuser 2015). That increases the extracellular glutamate concentrations and, in turn, excitability in the epileptic brain (Peterson and Binder 2020). GFAP increased expression was regarded as a pathological marker in PTZ-induced seizure experimental models (Ahmed et al. 2005). The current study revealed that EMPA pretreatments impacted neuroprotective effects in a dose-dependent manner by expressing less immunoreactive GFAP in the hippocampal tissues in comparison to the PTZ group, as this indicated a less inflammatory response and less brain injury. This may also be due to the anti-inflammatory effect of EMPA (Pirklbauer 2021).

Recently, studies have focused on studying certain molecules and their roles in epileptogenesis in order to understand new underlying mechanisms and seek new therapeutic strategies. Among these molecules, BDNF is considered one of the most recently studied factors. However, many debates have arisen on whether BDNF inhibits or promotes epileptogenesis (Wang et al. 2021). Here, results supported the hypothesis of targeting the BDNF-TrkB axis in the prevention of epilepsy. That was in alignment with the study that stated that mBDNF is considered the main functional isomer that is responsible for neuroprotective effects in the adult brain (Kowiański et al. 2018). In the current study, the PTZ group indicated an upregulation of proBDNF levels but a significant decrease in mBDNF expression in relation to its precursor proBDNF when compared to the control group. Interestingly, a study reported that a rapid increase in proBDNF following status epilepticus induced by pilocarpine may be in part due to decreasing its cleavage due to a decrease in tissue plasminogen activator and an increase in plasminogen activator inhibitor-1 (PAI-1), an inhibitor of extracellular and intracellular cleavage, and that proBDNF is initially enhanced in response to the acute phase of epileptogenesis (Thomas et al. 2016). These findings support our results and suggest that PTZ may also retard proBDNF activation, but this has to be further investigated. Despite increased proBDNF expression in EMPA treatment blots, EMPA revealed sufficient proteolytic cleavage of proBDNF to the mature form (mBDNF), which is suggested to play a role in alleviating neuronal apoptosis (Gerenu et al. 2017). That was indicated by the significant increase in relative mBDNF/proBDNF expression in EMPA treatments in comparison to the PTZ group. Unlike EMPA 1, the EMPA 3 group showed a significant increase in relative mBDNF/proBDNF expression in relation to PTZ-expressed levels, suggesting its potentiated neuroprotective effects when compared to the EMPA 1 group. As discussed, VPA exhibited powerful neuroprotective effects, with the most capability of preserving the normal hippocampal architecture being damaged by PTZ. In addition, the VPA group encouraged the BDNF pathway by showing the highest mBDNF/proBDNF expression among other treatment groups. This supported our postulation that there is a positive correlation between mature BDNF upregulation and neuroprotection. Moreover, research that studied the relationship between BDNF and oxidative stress indicated a direct interaction between them, where the decrease in BDNF expression increases the sensitivity to oxidative damage under stressful conditions. That may further explain the protective effects of EMPA against PTZ-induced oxidative stress (Hacioglu et al. 2016).

BDNF transcription is regulated by different transcriptional factors, including Npas4 and CREB, in response to different stimuli (Esvald et al. 2020). The transcription is stimulated as CREB is being phosphorylated and activated (Dyson and Wright 2016). BDNF-TrkB binding initiates the phosphorylation of CREB via the calcium/calmodulin-dependent kinase IV (CaMKIV)-regulated pathway and the Ras-dependent pathway (Finkbeiner et al. 1997). Therefore, BDNF was reported to increase pCREB levels (Xu et al. 2018). CREB is believed to be one of the transcriptional factors that is being induced by extracellular stimuli as in the case of prolonged seizures known as status epilepticus (Zhu et al. 2015). Moreover, it has been reported that the increase in excitatory neurotransmitters or intracellular calcium following the activation of voltage- or ligand-gated channels such as the NMDA receptor (glutamate receptor) is considered to be due to the different stimuli that activate the signaling process of CREB (Alberini 2009). Indeed, regarding its role in the potentiation of neuronal excitability in the hippocampus, CREB has been recently identified as a contributor to the pathogenesis of epilepsy (Wang et al. 2020). In this study, CREB levels (43 kDa) (Zhu et al. 2015) were notably reduced in treatment groups in comparison to the PTZ group, which showed markedly increased levels of CREB due to hyperexcitability and excessive depolarization. Additionally, the VPA group results were in concordance with a previous study that reported CREB downregulation after VPA treatment in pediatric epileptic patients (Floriano-Sánchez et al. 2018). Current results also revealed that the treatment groups reversed the significant upregulation of Npas4 enhanced by PTZ. In this context, it was reported that Npas4 expression is selectively enhanced by Ca2 + entry in the hippocampus in the PTZ-kindling model to maintain neuronal homeostasis through a negative feedback effect for the management of epilepsy as a defense mechanism following seizures to prevent hyperexcitability (Shan et al. 2018). Npas4 acts dependently on the regulation of the expression of different genes (e.g., BDNF), which in turn control the number of GABA-releasing synapses that form on excitatory neurons (Lin et al. 2008). However, the implication of Npas4, either directly or indirectly, in the propagation of inhibitory synapses is still controversial (Maya-Vetencourt 2013). Based on the previously mentioned findings, the downregulated levels of Npas4 in treatment groups may account for the decrease in the severity of seizures and hyperexcitability and influence their protective effects against PTZ insults. VPA has shown antiepileptic effects by interfering with postsynaptic Ca2 + influx (Franceschetti et al. 1986), so its significant inhibitory effect on Npas4 expression triggered by PTZ may be attributed to its action on calcium influx. Besides, no significant difference was observed between VPA and EMPA (3 mg/kg) regarding Npas4 contents; accordingly, the effect of EMPA on voltage-gated calcium channels has to be further studied. Understanding the Npas4/ CREB/ BDNF signaling pathways is considered controversial. More excitingly, recent researches have proposed their neuroprotective roles in cell survival and neuronal plasticity. Nevertheless, overexpression of these genes may be associated to deleterious effects and the triggering of various neurological disorders. Furthermore, prolonged activation of this pathway may result in neuronal loss especially in CA1 neurons and the propagation of epileptic seizures (Lopez de Armentia et al. 2007).

This research was the first to evaluate the antiepileptic effect of a drug based on Davoudi’s window-kindling model. More excitingly, we were eager to highlight new prospective mechanisms of EMPA, a hypoglycemic agent, in neurological studies seeking to improve the quality of life of epileptic patients. In view of aforementioned findings, EMPA was suggested to exert anticonvulsant effects against experimentally PTZ-induced seizures. This was illustrated by the significant improvement of biochemical markers, as it attenuated oxidative stress and mitochondrial damage (GSH and MDA) and reduced neuronal astrocytosis (GFAP) and apoptosis (BAX) induced by PTZ. Accordingly, seizure severity was reduced, and behavioral impairments were greatly improved. In addition, EMPA impacted neuroprotective effects by alleviating the neuronal loss in the hippocampal region, the marked dysregulation of Npas4 and CREB contents, and the enhancement of neuronal survival and plasticity, as evidenced by the encouragement of sufficient proteolytic cleavage of the precursor (proBDNF) and the formation of the mature (mBDNF) that binds to its high-affinity receptor (TrkB) to play a role in neurogenesis and prevention against the deteriorating effects of PTZ. However, the exact mechanism by which EMPA enhances the conversion of proBDNF to the mature form and its increased expression still needs to be elucidated.

### Limitations

The doses of EMPA used in the study were based on the clinically tolerable doses of EMPA as an oral hypoglycemic drug; however, the optimal dosage of EMPA as a neuroprotective agent has to be further studied. In addition, future studies may consider the combination of EMPA with the standard antiepileptic drugs to further validate the findings.

## Conclusion

In conclusion, empagliflozin may show prospective neuroprotective effects in different neurological manifestations, including epilepsy (Supplementary Fig. 1) However, further investigations concerning the exact mechanism of epileptogenesis and the implication of EMPA in the management of epileptic seizures are still needed.

## Electronic Supplementary Material

Below is the link to the electronic supplementary material.

Supplementary Material 1

## Data Availability

No datasets were generated or analysed during the current study.
