# Supplementary material for: Empagliflozin Mitigates PTZ-Induced Seizures in Rats: Modulating Npas4 and CREB-BDNF Signaling Pathway
Source: J Neuroimmune Pharmacol. 2025 Jan 7;20(1):5. doi: 10.1007/s11481-024-10162-6 (PMC11706855; doi:10.1007/s11481-024-10162-6)

**Supplementary Figure 1: Graphical Abstract**

**
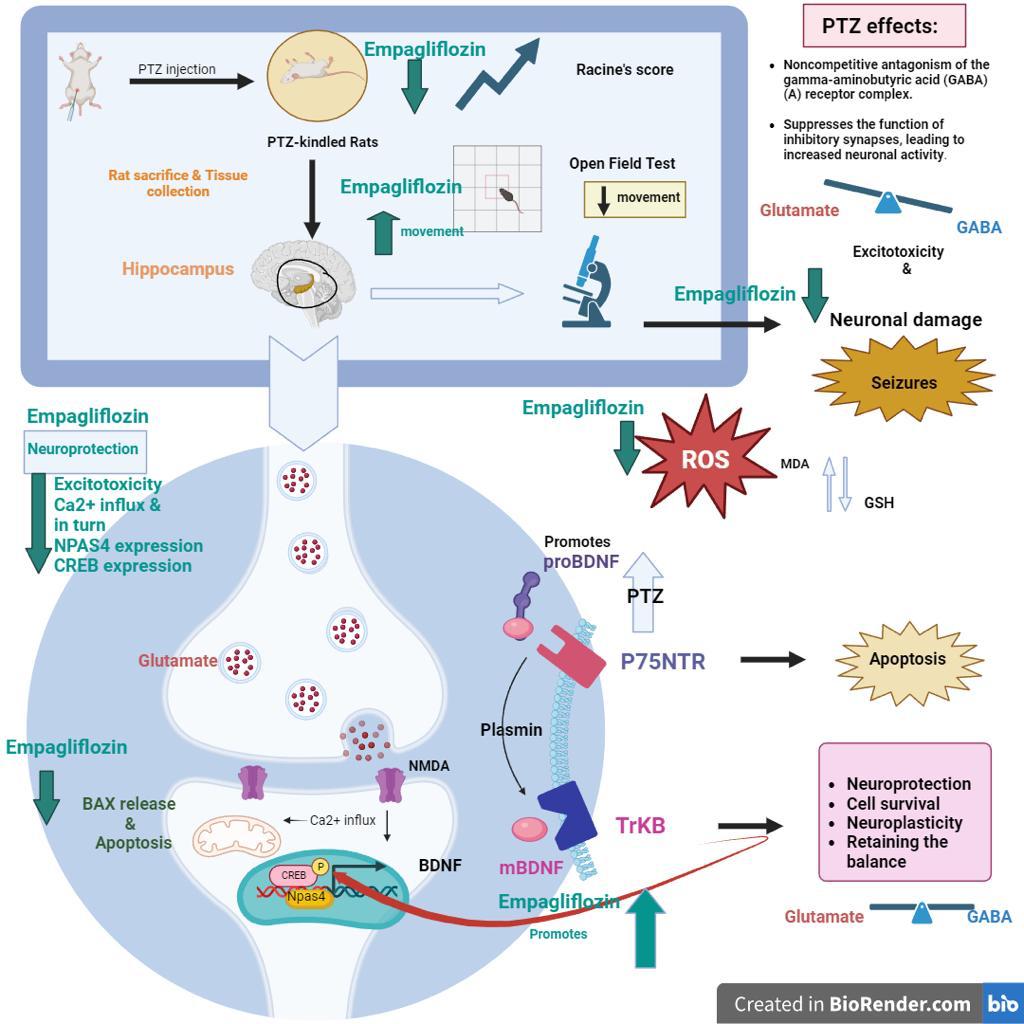
**

**Graphical Abstract: The effect of pretreatment with EMPA (1&3 mg/kg) against PTZ induced kindling epilepsy in rats.** * PTZ was intraperitoneally injected (37.5 mg/kg) where rats received only seven injections: 4 initial doses day after day followed by 3 consecutive injections at 29^th^, 31^th^ and 33^th^ days to induce seizures in rats. EMPA pretreatments were given at 1 and 3 mg/kg orally daily. Abbreviations: PTZ: pentylenetetrazole, EMPA: empagliflozin, win-: window.

**Supplementary Figure 2: Uncropped gels and blot images**


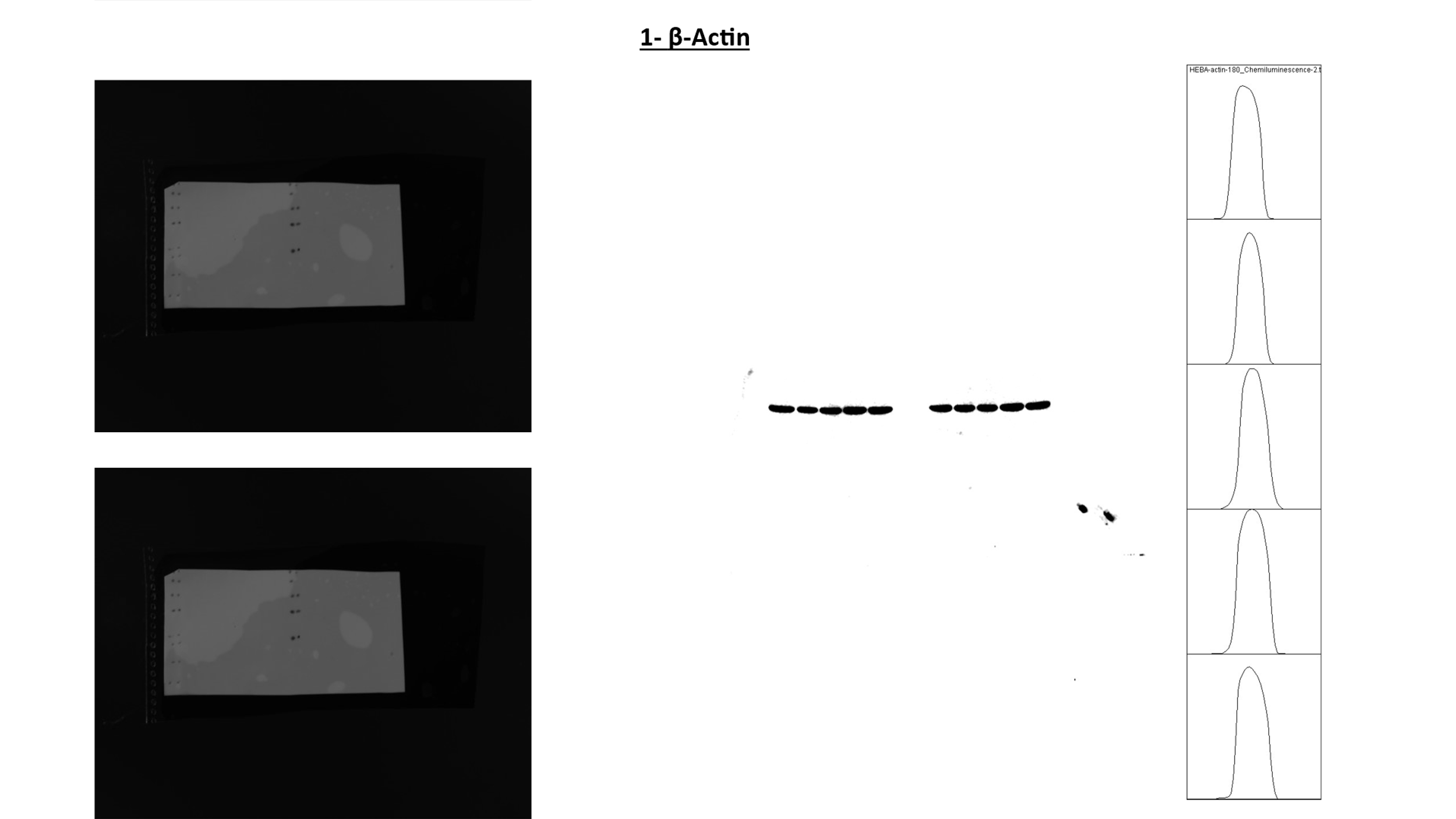


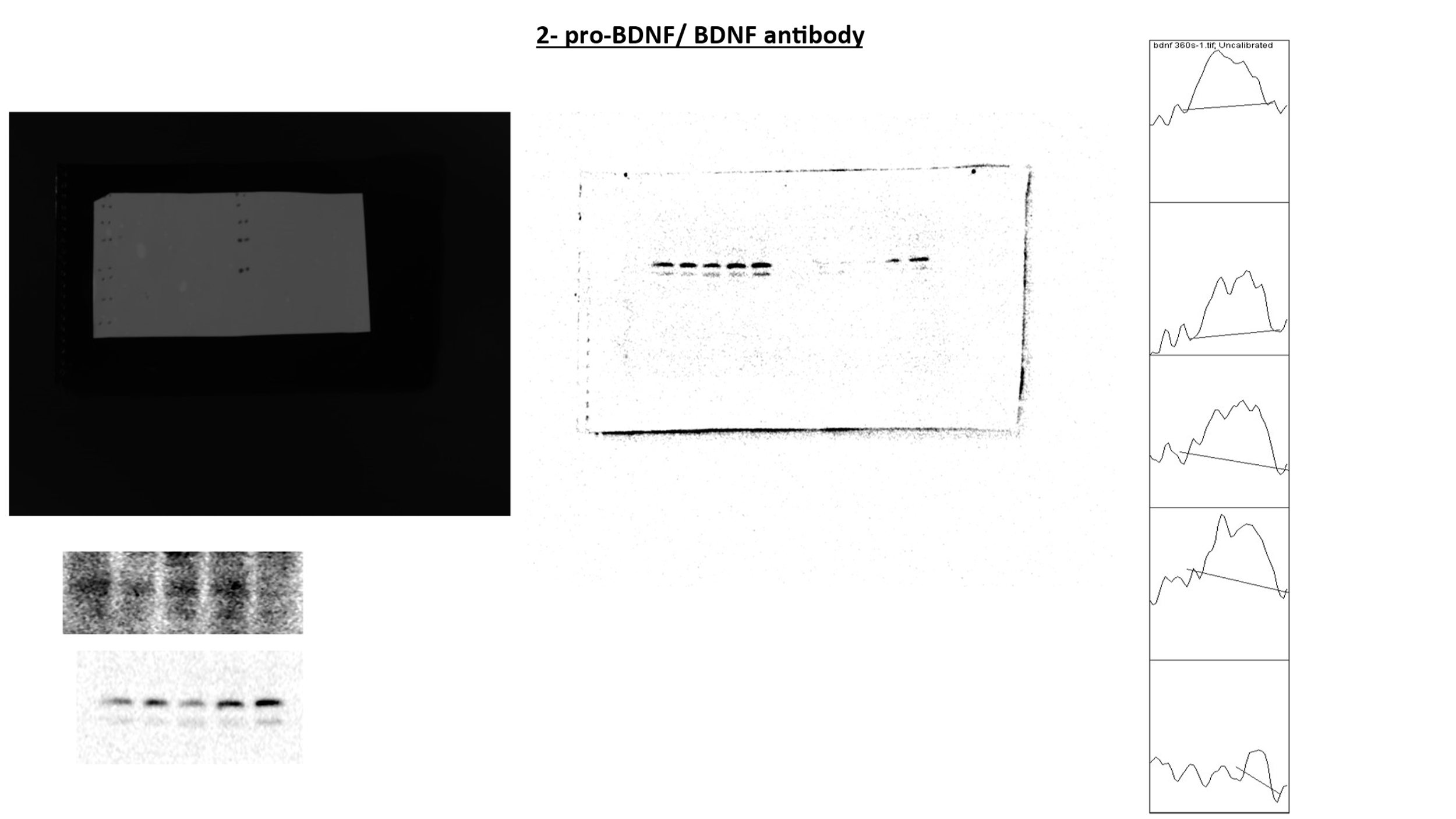


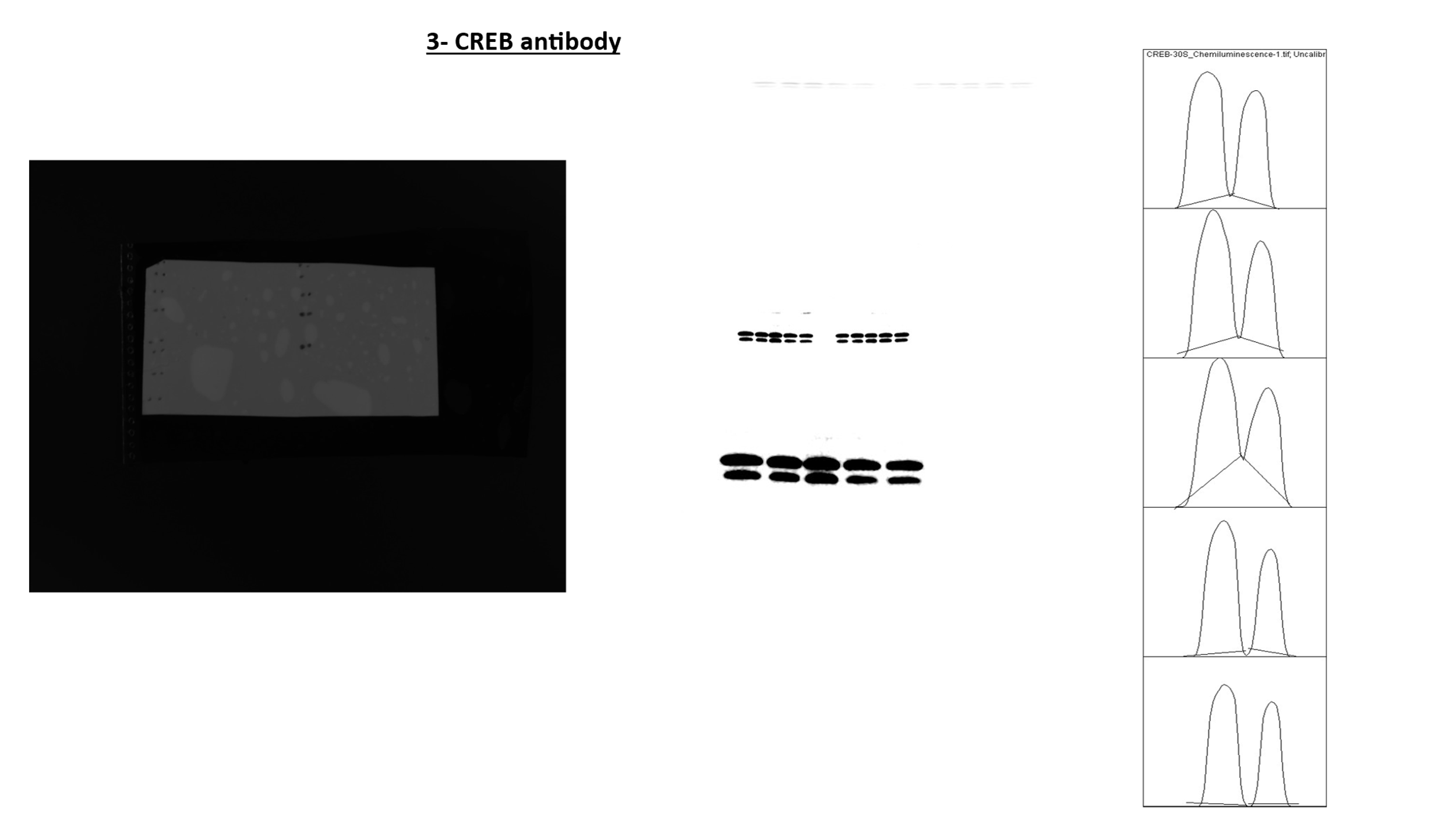

Supplement: Supplementary file 1 — Supplementary Material 1 [file 11481_2024_10162_MOESM1_ESM.docx]
